# Fresh Agricultural Products Supply Chain Coordination and Volume Loss Reduction Based on Strategic Consumer

**DOI:** 10.3390/ijerph17217915

**Published:** 2020-10-28

**Authors:** Fang Qiu, Qifan Hu, Bing Xu

**Affiliations:** 1 School of Management, Nanchang University, Nanchang 330031, China; 352204218001@email.ncu.edu.cn; 2 School of Business Administration, Nanchang Institute of Technology, Nanchang 330099, China; setsail@nit.edu.cn

**Keywords:** fresh agricultural products, strategic consumers, supply chain coordination, volume loss

## Abstract

The reduction of fresh agricultural product volume loss throughout the supply chain system is of high importance due to their perishable nature and impact on society, the economy, and environment. In this paper, three models for two-stage pricing, coordination, and volume loss reduction of the supply chain where third-party logistics service providers and retailers act as a Stackelberg leader and a follower for fresh agricultural products are developed, taking into account both volume loss during transport and quality loss in retail in the presence of strategic consumers. The following results are drawn from the contract for sharing revenues and service costs: (1) The supply chain achieve coordination and the products are healthier for consumers; (2) the coordination leads to a reduction in the three types of volume losses simultaneously only if the lowest marginal costs of the supply chain occur under certain conditions; and (3) the increase in the service sensitivity coefficient, the increase in the freshness discount coefficient under certain conditions, the decrease in the consumer benefit discount coefficient under certain conditions, and the decrease in the price sensitivity coefficient lead to an increase in the profit of the supply chain and a reduction in the three types of volume losses.

## 1. Introduction

**Figure 1:**
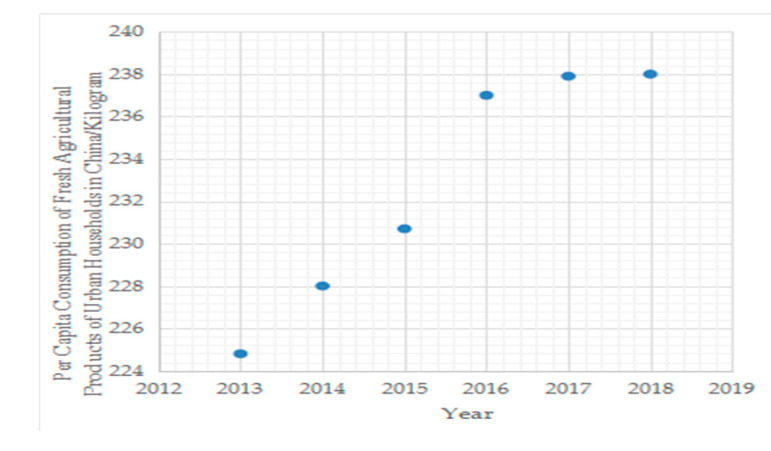
Per capita consumption of fresh agricultural products of urban households in China since 2013.

Fresh agricultural products normally include vegetables, fruits, livestock, poultry, seafood, eggs, milk, and meat [1] which are key to improving people’s quality of diet. According to these categories that include fresh agricultural products and the per capita consumption of Major Foods of Urban Households of the *2019 China Statistical Yearbook*, it is obvious that the per capita consumption of fresh agricultural products of Urban Households in China has increased since 2013 from Figure 1. With the pursuit of health, fresh product supermarkets have sprung up to meet consumer demand. Therefore the pricing of the these products has received attention from retailers and researchers [2,3,4,5]. There are about 20–30% of fresh agricultural products in China wasted in different stages of the supply chain [6,7]. Therefore, fresh agricultural supply chains with third-party logistics service providers (TPLSP) that offer refrigeration services and transport has gained much attention due to their characteristics of deterioration [2,4,8,9,10]. In addition, in practice, transport logistics is often outsourced to TPLSP with less quantity and quality loss of fresh agricultural products. The deterioration of fresh agricultural products includes the loss of quality where the quality reduces over time as well as physical volume loss in which some portion of items are damaged over time [6]. There are a few models taking into account both volume loss and quality loss [2,4,7,8,9,11]. However, the quality of products is assumed to be unchanged during retail in the studies of Yu et al. [2,4] and Cai et al. [8], however physical volume loss during transport is not considered in the studies of Qin et al. [11]. The cold chain services is not the decision variable which is related to both volume loss and quality loss in the study of Yan et al. [7] and consumers’ strategic behavior is not taken into account in all these studies. Both the volume loss during transport and quality loss during the retail of fresh agricultural products and consumer strategic behavior are simultaneously taken into account in this paper, unlike the above studies.

Fresh agricultural products on shelves become unattractive to consumers because of quality loss, thus some firms use a dynamic pricing strategy which is a common mechanism for selling perishable products [12] to attract consumers to buy in order to obtain greater profit [13]. Fresh fruits and vegetables in FRESHIPPO are sold at a discount every night. However, consumers are becoming increasingly rational when facing price changes [14]. The strategic consumer will adjust their buying strategies based on price changes [14]. If sellers ignore the presence of strategic consumers, they will lose profits [14,15] therefore, the retailer of fresh agricultural products must consider the waiting behavior of these strategic consumers when making a pricing decision because of the quality loss of products during retail. Most model studies about pricing in the presence of strategic consumers are one-echelon supply chain with one monopoly firm or oligopolistic competition firm. There are few models that study a decentralized supply chain in the presence of strategic consumers [16,17]. Even in the study of them, the value reduction and volume reduction of the products are not taken into account. While the value reduction and volume reduction of the products are taken into account in the two-echelon supply chain model based on strategic consumers in this paper.

Some 1.3 billion tons of food are wasted or lost globally per year [18] and the statistics show that about 80 million tons of fresh agricultural products in China are wasted per year [7]. Additionally, there is about 4.3% of the vegetables and fruit which is delivered to Swedish retailers wasted during delivery or at the store [19]. Food wastage or loss impacts society, the economy, and environment [20,21]. Therefore, food loss and food waste reduction at one or more stages of the food chain are further studied by many researchers through their empirical study [21] and case study analysis [22,23]. Most of these studies are qualitative analysis. Only Mohammadi et al. [6] found that the percentage of product waste can be reduced through fresh–product supply chain coordination based on the mathematical model with the equilibrium theory and backward induction method. The mathematical model with the backward induction method reflects the pull of consumer demand on the supply chain directly, and the quantitative relationship between each node of the supply chain is obvious. However, only a percentage of product reduction is analyzed and the impacts of total demand or profit on product waste are not taken into account [6]. The total carbon emission is analyzed in the study of Bai et al. [24]. Therefore, the total demand volume loss (*tdl*) and the per unit profit volume loss (*ppl*) are important too for decision makers. Then the reduction of the three types of volume losses including per unit demand volume loss (*pdl*), per unit profit volume loss (*ppl*), and total demand volume loss (*tdl*) are analyzed in this paper based on the mathematical model with the equilibrium theory, Stackelberg game, and backward induction method in this paper.

Considering the above considerations, this paper will aim to answer the following three questions through the mathematical model with the equilibrium theory, Stackelberg game, and backward induction method: (1) Can the two-echelon supply chain where the TPLSP and retailer act as a Stackelberg leader and a follower for fresh agricultural products achieve coordination when both the volume loss during transport and quality loss during retail in the presence of strategic consumers be taken into account? Can fresh agricultural products be more beneficial for health? (2) Can the coordination lead to a reduction in the three types of volume losses (per unit demand volume loss, per unit profit volume loss, and total demand volume loss)? (3) What is the impact of consumer strategic behavior on the profit, coordination of the chain, and optimal logistic level when strategic consumers extend to the two-echelon supply chain where the TPLSP and retailer are engaged in after its coordination? Therefore, the considerations undertaken in this article will fill the gap in the literature through the following these two aspects: (1) Both the volume loss during transport and the quality loss during retail of the fresh agricultural products and consumer strategic behavior are taken into account in the supply chain model and (2) three types of volume losses and their reductions are analyzed based on the mathematical model.

The remainder of this paper is organized as follows. In Section 2, we provide the literature review. In Section 3, we develop the centralized model, decentralized model, and contractual cooperation model. The decentralized model and contractual cooperation model are compared. In Section 4, we present numerical analysis. The result is discussed in Section 5. Conclusions and future research plans are presented in Section 6. All proofs are found in Appendix B and the decentralized model can be found in Appendix A.

## 2. Literature Review

There are three streams of literature related to this paper. The first stream focuses on the supply chain and coordination for deteriorating items. A fresh agricultural product has the same characteristic as deteriorating items as the time goes on [10]. Most studies describe the characteristics of deteriorating items under three different modes: (1) Quality deterioration [25,26,27,28,29,30], (2) the physical quantity deterioration [31,32,33,34,35] and, (3) both quantity and quality deterioration [2,4,7,8,9,11]. Both quality loss and physical volume loss include the loss at any stage of the supply chain. There are a few models that take into account both the quality and physical volume loss [2,4,7,8,9,11]. While the quality during retail is assumed to be unchanged in the studies of Yu et al. [2,4], Cai et al. [8], and Wu et al. [9], both the quality and physical volume loss during retail and not transport are taken into account in the study of Qin et al. [11]. Although both the volume loss during transport and the quality loss during retail of the fresh agricultural products are taken into account in the study of Yan et al. [7], the cold chain services level assumed to be related to both loss is not the decision variable. The consumers’ strategic behavior is not taken into account in all these studies, unlike this paper. Many studies make the decision of the freshness-keeping effort/preservation technology investment whose purpose is to maintain quality and reduce the volume loss rate during the transport of fresh products [2,4,6,9,10,36] or only reduce volume loss rate during storage [32,33,35], so the revenue and cost sharing contract or revenue sharing and cooperative investment contract is designed to coordinate the two-echelon [6,9,32,33,35,36] or three-echelon [10] supply chain in order to cut down on the influence of the double marginalization effect. Gu et al. [37] find that both the buyback contract and a revenue- and cost-sharing contract could coordinate the supply chain consisting of one fresh product supplier and one e-retailer. Differing from all these papers, both the volume loss during transport and the quality loss during the retail of fresh agricultural products and consumer strategic behavior are simultaneously taken into account in this paper, and a two-stage pricing decision is made.

Another stream of research focuses on dynamic pricing for deteriorating items and devalued products in the presence of strategic consumers. These studies can be divided into two categories: (1) A dynamic pricing model for a one-echelon supply chain with a monopoly (monopolist) firm [13,14,15,38,39,40,41,42] or oligopolistic competition firms [43,44,45] in the presence of strategic consumers, and (2) a dynamic pricing model for a decentralized supply chain in the presence of strategic consumers [16,17]. The presence of strategic consumers is detrimental to the retailer [38] and the seller would lose profits without paying attention to the presence of strategic consumers [14,15,39,43]. Considering the presence of strategic consumers, posterior price matching and delay posterior price matching strategies are compared, with the best choice for the seller is related to hassle cost and valuation differences between high- and low-end consumers [40]. Similarly, the preannounced pricing policies and responsive pricing are compared, preannounced pricing policies can be more advantageous to the seller [15] however, responsive pricing can be better to the seller in the presence of social learning consumers [41]. Enhanced design and quick responses can mitigate strategic behavior, and the complementarity of combining them in a fast fashion system is strongest when consumers are very strategic [42]. Contrary to previous studies, strategic consumers may yield increased revenues in specific scenarios when the seller has the ability of quick response [13]. Other researchers have developed the dynamic pricing model for oligopolistic competition firms in the presence of strategic consumers [43,44,45]. In addition, a low-quality firm suffers substantially more than a high-quality firm when customers become more strategic under competition [44]. When strategic consumers extend to a decentralized supply chain [16,17], the profit of a centralized structure with one seller may not always gain more than a decentralized structure with a two-echelon supply chain in the presence of strategic consumers because of the reference price effect [17] or quantity commitment and price commitment [16]. However in a study of them, the value reduction and volume reduction of the products are not taken into account. There are some differences between this stream of literature and this paper. Firstly, similarly to some of the above studies [14,15,38], this paper considers the decrease of quality by the freshness level discount coefficient, and the value of the product is related to the logistics service level provided by TPLSP. Secondly, this paper explores the impact of consumer strategic behavior when strategic consumers extend to the two-echelon supply chain where the TPLSP and retailer are engaged in, which means that this paper considers the impact of consumer strategic behavior on not only the profit of fresh agricultural products supply chain (FASC), but also three types of volume loss and logistic service level.

The last stream of this paper is volume loss reduction. Volume loss occurring during transport in this paper is a subset of food waste and food loss which occur at any stage of the food supply chain [46]. According to Food and Agriculture Organization of the United Nations (FAO) [47], food loss and food waste are losses occurring during the different food supply chain stages, whereby food loss occurs from agriculture up to industrial transformation, while food waste occurs during the final retail and consumption stages [48,49]. Another definition is that food waste can be divided into inedible part and food loss [50]. From this definition, food loss is a subset of food waste [49,51]. Food wasted or lost impact society, the economy, and environment [20,21,52,53]. Therefore, food loss and food waste reduction are further studied by many researchers [21,22,23,48,51,52,53,54]. Food loss and food waste can be reduced through implementing the ISO 22000 standard [21], adopting the circular economy approach [23], identifying the driving power of challenges inhibiting the reduction of waste [51], communication and cooperation of the member along the chain [52], stronger government regulation and intervention [53], demand and shelf-life information sharing in fresh food supply chains [54] and other specific methods for each stage of the food chain which are summarized in the study of Prinsipoto et al. [23] and Kummu et al. [48]. These measures are obtained from empirical study, case study analysis, review, and other qualitative analysis methods. Only Mohammadi et al. [6] found that the percentage of product waste can be reduced through fresh–product supply chain coordination based on the mathematical model with equilibrium theory and backward induction method. However they analyze only one type of volume loss. The three types of volume losses including reduction are analyzed based on the mathematical model with the equilibrium theory, Stackelberg game, and backward induction method in this paper.

## 3. Problem Description and Models

### 3.1. Problem Description

**Figure 2:**
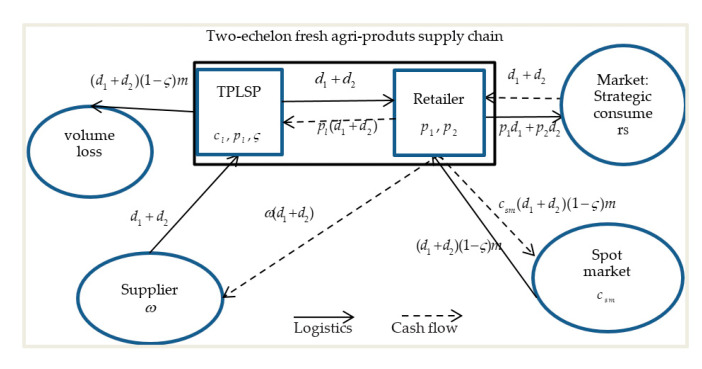
The two-echelon fresh agri-products supply chain.

A retailer procures fresh agricultural products at a wholesale price *ω* and ships them through a TPLSP whose logistics service level ς impact volume loss and quality loss with a unit cost *c_l_* and a unit logistics service price *p_l_* charged (to the retailer) [2,8,36] to a distant market where the retailer sells the product to strategic consumers with a preannounced two-stage pricing strategy *p_i_* (*i* = 1,2) where *p*_2_ < *p*_1_ [3]. Due to volume loss during transportation, the retailer procures the same product from the spot market providing adequate fresh agricultural products to replace the ones that deteriorated during transport at a price *c_sm_* with the benefit of more information about the market before or during selling, which is similar to the assumption in the paper by Yu et al. [2]. In the two-echelon supply chain, the TPLSP and retailer are risk-neutral and act as a Stackelberg leader and a follower such as the large logistics company Maersk and their relatively small client [9]. The TPLSP acts as a Stackelberg leader too in the paper of Cai et al. [8]. The structure of the problem is described in Figure 2.

The total market size is normalized to 1 [17,55]. Strategic consumers make purchases between the two periods of the sales horizon to maximize their expected utilities. It is assumed that fresh agricultural products are completely available to all buying customers [17]. So for a strategic consumer, the utility of buying an item in the second period is discounted by a homogeneous discount factor *δ*, which can be interpreted as the level of strategic behavior [16,17,43]. Similar to Yu et al. [2], we assume that the freshness level function in period one is $\theta_{1}=h\varsigma$ [2], where *h* represents the sensitivity of the freshness level in period one to the logistics service level. Thus, the freshness level function in period 2 is $\theta_{2}=\gamma h\varsigma$, where *γ* ∈ (0,1) represents the discount factor of the freshness level in period two compared to period one. Then, similar to Zheng et al. [28] and Hu et al. [56], the utility functions of consumers in two periods are:

(1) $$u_{i}=\left\{ \begin{array}{l} v-\rho p_{1}+\kappa \theta_{1}\\ \delta (v-\rho p_{2}+\kappa \theta_{2}) \end{array}\right.\begin{array}{cc} = & \left\{ \begin{array}{l} v-\rho p_{1}+\varepsilon \varsigma \begin{array}{cc} & (i=1) \end{array}\\ \delta (v-\rho p_{2}+\varepsilon \gamma \varsigma )\begin{array}{cc} & \begin{array}{cc} & \left(i=2\right) \end{array} \end{array} \end{array}\right. \end{array}$$

where *v* represents the consumers’ basic cognitive value of fresh agricultural products following a uniform distribution of [0,1] [17,28], and *ρ* and *k* represent the measures of the sensitivity of the utility of consumers to the selling price and freshness level, respectively. In addition, *ε* = *kh* represent the measure of the sensitivity of the utility of consumers to the logistics service level [2]. Since strategic consumers decide the purchase stage by maximizing utility and non-negative utility, the demands of the two periods are:

(2) $$d_{i}=\left\{ \begin{array}{l} \frac{1-\rho p_{1}+\varepsilon \varsigma -(\varsigma \gamma \varepsilon -\rho p_{2}+1)\delta }{1-\delta }\begin{array}{cc} & \left(i=1\right) \end{array}\\ \frac{\rho (p_{1}-p_{2})-\varepsilon \varsigma +\varsigma \gamma \varepsilon }{1-\delta }\begin{array}{ccc} & & \left(i=2\right) \end{array} \end{array}\right.$$

according to the actual situation, the demand in the second stage is positive ($d_{2}>0$).

The demands of the two periods above are based on the assumption that consumers’ basic cognitive value of fresh agricultural products follows a uniform distribution of [0,1]. The more general case is that all customers’ basic cognitive value of fresh agricultural products *v* share a common probability density function (pdf) *f(x)* and cumulative distribution function (cdf) *F(x)*. Then the mathematical model will be too complex to get the Equilibrium solution. Therefore, for mathematical simplicity and without loss of generality, consumers’ basic cognitive value following a uniform distribution of [0,1] is assumed. This assumption is widely accepted in the literature where the deterministic demand are obtained from the consumers’ utility functions [15,17,28,44]. Then the baseline results for further analyzing and comparing are gained based on this assumption. Additionally, the managerial implications obtained from the baseline results can offer initial suggestions to the practice of supply chain members.

We summarize all notations used through the paper in Table 1.

In the equations that follow, the superscript symbol “*” represents the optimal decisions, the superscript symbol “*C*” represents the centralized model, the superscript symbol “*tr*” represents the decentralized model, and the superscript symbol “*cc*” represents contractual cooperation model. To simplify the calculation process, we denote $\alpha =2\rho mc_{sm}+(1+\gamma )\varepsilon$, $\beta =1-(mc_{sm}+c_{l}+\omega )\rho$.

### 3.2. Models

#### 3.2.1. Centralized Model

Assume that the TPLSP and retailer make decisions together with the goal of maximizing the profit of FASC, based on the above assumptions, the profit of FASC is:

(3) $$\pi_{sc}^{C}(p_{1}^{C},p_{2}^{C},\varsigma^{C})=(p_{1}^{C}-\omega )d_{1}^{C}+(p_{2}^{C}-\omega )d_{2}^{C}-c_{sm}(1-\varsigma^{C})m(d_{1}^{C}+d_{2}^{C})-c_{l}(d_{1}^{C}+d_{2}^{C})-\tau {\left(\varsigma^{C}\right)}^{2}/2.$$

**Proposition** **1.** *Under the conditions L_i_ (i = A,B), the optimal prices in two periods and the optimal logistics service level in the centralized model are*:

(4) $$\left\{ \begin{array}{l} p_{1}^{C*}=\frac{1}{\rho }-\frac{\beta [2(1-\delta^{2})\tau \rho -(1-\delta )\alpha (\gamma +1)\varepsilon -{\left(\gamma -1\right)}^{2}\varepsilon^{2}(1+\delta )]}{\{(\delta +3)[2\tau \rho (1-\delta )-{\left(\gamma -1\right)}^{2}\varepsilon^{2}]-\alpha^{2}(1-\delta )\}\rho }\\ p_{2}^{C*}=\frac{1}{\rho }-\frac{2\beta [2\tau \rho (1-\delta )-{\left(\gamma -1\right)}^{2}\varepsilon^{2}-(1-\delta )\alpha \gamma \varepsilon ]}{\{(\delta +3)[2\tau \rho (1-\delta )-{\left(\gamma -1\right)}^{2}\varepsilon^{2}]-\alpha^{2}(1-\delta )\}\rho }\\ \varsigma^{C*}=\frac{2(1-\delta )\alpha \beta }{\left(\delta +3)[2\tau \rho (1-\delta )-{\left(\gamma -1\right)}^{2}\varepsilon^{2}]-\alpha^{2}(1-\delta \right)} \end{array}\right..$$

The value of *L_i_* (*i = A,B*) and the proof of Proposition A2 are shown in the Appendix B.

Substitute Equation (4) into the demand function (2) and profit function (3) of FASC, the demand and profit are:

(5) $$\left\{ \begin{array}{l} d_{1}^{C*}=\frac{\beta [2\tau \rho (1-\delta )-{\left(\gamma -1\right)}^{2}\varepsilon^{2}-(\gamma -1)\alpha \varepsilon ]}{\left(\delta +3)[-{\left(\gamma -1\right)}^{2}\varepsilon^{2}+2\tau \rho (1-\delta )]-\alpha^{2}(1-\delta \right)}\\ d_{2}^{C*}=\frac{\beta [2\tau \rho (1-\delta )-{\left(\gamma -1\right)}^{2}\varepsilon^{2}+(\gamma -1)\alpha \varepsilon ]}{\left(\delta +3)[-{\left(\gamma -1\right)}^{2}\varepsilon^{2}+2\tau \rho (1-\delta )]-\alpha^{2}(1-\delta \right)}\\ d^{C*}=\frac{2\beta [2\tau \rho (1-\delta )-{\left(\gamma -1\right)}^{2}\varepsilon^{2}]}{\left(\delta +3)[-{\left(\gamma -1\right)}^{2}\varepsilon^{2}+2\tau \rho (1-\delta )]-\alpha^{2}(1-\delta \right)}\\ \pi_{sc}^{C*}=\frac{\beta^{2}[2\tau \rho (1-\delta )-{\left(\gamma -1\right)}^{2}\varepsilon^{2}]}{\{(\delta +3)[-{\left(\gamma -1\right)}^{2}\varepsilon^{2}+2\tau \rho (1-\delta )]-\alpha^{2}(1-\delta )\}\rho } \end{array}\right..$$

From the conditions and the above formula, the following Proposition 2 is obtained.

**Proposition** **2.** *In a centralized model under the conditions of L_i_ (i = A,B): (1) The marginal profit of FASC is* β/(2ρ) *;(2) the total demand and the profit of FASC are inversely proportional to the consumer utility discount coefficient respectively; (3) if* ${\left(1-\delta \right)}^{2}\tau \rho >2{\left(\gamma -1\right)}^{2}\varepsilon^{2}$ *, the optimal logistics service level is inversely proportional to the consumer utility discount coefficient, while the per unit demand volume loss and the per unit profit volume loss are directly proportional to it otherwise, the optimal service level is directly proportional to it, while the per unit demand volume loss and per unit profit volume loss are inversely directly proportional to it; (4) the optimal service level, the total demand, and the profit of FASC are directly proportional to the service sensitivity coefficient, while the per unit demand volume loss and per unit profit volume loss are inversely proportional to it; (5) the total demand and profit of FASC are directly proportional to the freshness level discount coefficient; (6) if* $(\delta +3)[2\tau \rho (1-\delta )-2\alpha (1-\gamma )\varepsilon -{\left(\gamma -1\right)}^{2}\varepsilon^{2}]+\alpha^{2}(1-\delta )<0$ *, the optimal service level is inversely proportional to the freshness level discount coefficient, while the per unit demand volume loss and per unit profit volume loss are directly proportional to it otherwise, the optimal service level is directly proportional to the freshness level discount coefficient, while the per unit demand volume loss and per unit profit volume loss are inversely proportional to it; (7) if* $(\delta +3)[{\left(\gamma -1\right)}^{2}\varepsilon^{2}-2\tau \rho (1-\delta )]+\alpha^{2}(1-\delta )+4\alpha \beta (1-\delta )>0$ *, the total demand is directly proportional to the natural volume loss rate otherwise, the total demand is inversely proportional to it; (8) the profit of FASC is inversely proportional to the natural volume loss rate; (9) if* $\{(\delta +3)[{\left(\gamma -1\right)}^{2}\varepsilon^{2}-2\tau \rho (1-\delta )]+\alpha^{2}(1-\delta )\}(2\beta -\alpha )-4(1-\delta )\beta \alpha^{2}<0$ *, the optimal logistics service level is directly proportional to the natural volume loss rate otherwise, the optimal logistics service level is inversely proportional to it; and (10) the optimal logistics service level, the total demand, and the profit of FASC are inversely proportional to the price sensitivity coefficient, while the per unit demand volume loss and the per unit profit volume loss are directly proportional to it.*

The proof of Proposition A3 is shown in the Appendix B.

The following results are shown from Proposition 2 under the conditions of *L_i_* (*i = A,B*): (1) If 0<δ<G(γ) where $G(\gamma )=1-\sqrt{2/\left(\tau \rho \right)}(1-\gamma )\varepsilon$, then $pdl^{C*}$ and $ppl^{C*}$ increase with *δ*; if 1>δ>G(γ), then $pdl^{C*}$ and $ppl^{C*}$ decrease with *δ*. (2) If $2{\left(c_{sm}+\varepsilon \right)}^{2}+\tau \rho (\delta -1)>0$ and 0<γ<H(δ) where $H(\delta )=\left\{-(2\varepsilon +4mc_{sm})+\sqrt{\left(\delta +3)[4{\left(c_{sm}+\varepsilon \right)}^{2}+2\tau \rho (\delta -1)\right]}\right\}/2\varepsilon$, then $pdl^{C*}$ and $ppl^{C*}$ increase with *γ*; and if $2{\left(c_{sm}+\varepsilon \right)}^{2}+\tau \rho (\delta -1)>0$ and 1>γ>H(δ), then $pdl^{C*}$ and $ppl^{C*}$ decrease with *γ*; if $2{\left(c_{sm}+\varepsilon \right)}^{2}+\tau \rho (\delta -1)<0$, then $pdl^{C*}$ and $ppl^{C*}$ decrease with *γ*.

#### 3.2.2. Contractual Cooperation Model

We can obtain $\pi^{C*}-\pi^{tr*}>0$ because of:

(6) $$\pi_{sc}^{C*}-\pi_{sc}^{tr*}=(\delta +3)\beta^{2}\frac{\alpha^{2}{\left(\gamma -1\right)}^{4}\varepsilon^{4}+4\tau (1-\delta )\rho \{\tau \rho (\delta +3)2\tau \rho (1-\delta )-[\tau \rho (\delta +3)-\alpha^{2}]{\left(\gamma -1\right)}^{2}\varepsilon^{2}\}}{{\left[\alpha^{2}-4(\delta +3)\tau \rho \right]}^{2}(1-\delta )\{(\delta +3)[2\tau \rho (1-\delta )-{\left(\gamma -1\right)}^{2}\varepsilon^{2}]-(1-\delta )\alpha^{2}\}\rho },$$

under the conditions of *L_i_* (*i* = *A,B*) and *L_j_* (*j* = *C,D*) ($\pi^{tr*}$ and *L_j_* (*j* = *C,D*) is shown in Appendix A ‘Decentralized Model’). Which is mainly due to the existence of dual marginal utility, so the revenue and service-cost sharing contract $\left(\lambda ,\mu ,p_{l}^{cc}\right)$ is designed for supply chain coordination. The retailer pays the TPLSP’s logistics service price $p_{l}^{cc}$ to the TPLSP, and shares (1−λ) of its revenue with the TPLSP, simultaneously paying (1−μ) of the TPLSP’s service cost in order to encourage the TPLSP to improve its logistics service level. Under this contract $\left(\lambda ,\mu ,p_{l}^{cc}\right)$, the profit functions of the retailer and the TPLSP are:

(7) $$\pi_{r}^{cc}(p_{1}^{cc},p_{2}^{cc})=(p_{1}^{cc}d_{1}^{cc}+p_{2}^{cc}d_{2}^{cc})\lambda -(p_{l}^{cc}+\omega )(d_{1}^{cc}+d_{2}^{cc})-c_{sm}(d_{1}^{cc}+d_{2}^{cc})(1-\varsigma^{cc})m-(1-\mu )\tau {\left(\varsigma^{cc}\right)}^{2}/2,$$

(8) $$\pi_{l}^{cc}(\varsigma^{cc})=(p_{l}^{cc}-c_{l})(d_{1}^{cc}+d_{2}^{cc})-\mu \tau {\left(\varsigma^{cc}\right)}^{2}/2+[p_{1}^{cc}d_{1}^{cc}+p_{2}^{cc}d_{2}^{cc}](1-\lambda ).$$

The TPLSP and retailer act as a Stackelberg leader and a follower respectively, they aim to maximize their own profit. So the decentralized model is a bilevel programming problem which can be stated as:

(9) $$\left\{ \begin{array}{l} \left(\varsigma^{cc*})\in \arg\max\pi_{l}^{cc}(\varsigma^{cc},p_{l}^{cc},\lambda ,\mu ,p_{1}^{cc}(\varsigma^{cc}),p_{2}^{cc}(\varsigma^{cc})\right)\\ s.t.\begin{array}{cc} & \left(p_{1}^{cc}(\varsigma^{cc}),p_{2}^{cc}(\varsigma^{cc}))\in \arg\max\pi_{r}^{cc}(p_{1}^{cc},p_{2}^{cc},p_{l}^{cc},\varsigma^{cc},\lambda ,\mu \right) \end{array} \end{array}\right..$$

According to the above game order, we can obtain the following Proposition 3.

**Proposition** **3.** *Under the conditions of L_i_ (i = C,D) and L_j_ (j = C,D), we can obtain that the coordination of FASC can be realized with the revenue and service-cost sharing contract if:*

(10) $$\left\{ \begin{array}{l} 0<\lambda^{*}<1\\ \mu^{*}=\frac{\varepsilon [mc_{sm}{\left(\gamma -1\right)}^{2}\varepsilon +\tau (\gamma +1)(1-\delta )](1-\lambda^{*})}{(1-\delta )\alpha \tau }\\ p_{l}^{cc*}=c_{l}-(1-\lambda^{*})\frac{\left\{(\delta +3)[2(1-\delta )\tau \rho -\varepsilon^{2}{\left(\gamma -1\right)}^{2}]-(1-\delta )\alpha^{2}-2(1-\delta )\alpha \beta \}(1-\beta )+2(1-\delta )\alpha \beta (\omega +c_{l}\right)}{\{(\delta +3)[2(1-\delta )\tau \rho -\varepsilon^{2}{\left(\gamma -1\right)}^{2}]-(1-\delta )\alpha^{2}\}\rho } \end{array}\right.;$$

*the coordination of FASC can be perfectly realized if:*

(11) $$\left\{ \begin{array}{l} 1-N\frac{\left(\delta +3)[2(1-\delta )\tau \rho -\varepsilon^{2}{\left(\gamma -1\right)}^{2}]-\alpha^{2}(1-\delta \right)}{M[4(\delta +3)\rho \tau -\alpha^{2}](1-\delta )}<\lambda^{*}\\ \lambda^{*}<1-\tau \rho \frac{2{\left\{(\delta +3)[2(1-\delta )\tau \rho -\varepsilon^{2}{\left(\gamma -1\right)}^{2}]-(1-\delta )\alpha^{2}\right\}}^{2}}{M}\\ \mu^{*}=\frac{\varepsilon [mc_{sm}{\left(\gamma -1\right)}^{2}\varepsilon +\tau (\gamma +1)(1-\delta )](1-\lambda^{*})}{(1-\delta )\alpha \tau }\\ p_{l}^{cc*}=c_{l}-(1-\lambda^{*})\frac{\left\{(\delta +3)[2(1-\delta )\tau \rho -\varepsilon^{2}{\left(\gamma -1\right)}^{2}]-(1-\delta )\alpha^{2}-2(1-\delta )\alpha \beta \}(1-\beta )+2(1-\delta )\alpha \beta (\omega +c_{l}\right)}{\{(\delta +3)[2(1-\delta )\tau \rho -\varepsilon^{2}{\left(\gamma -1\right)}^{2}]-(1-\delta )\alpha^{2}\}\rho } \end{array}\right.,$$

*where*,

(12) $$M=\{(\delta +3)[2(1-\delta )\tau \rho -\varepsilon^{2}{\left(\gamma -1\right)}^{2}]-(1-\delta )\alpha (1+\gamma )\varepsilon \}[2(1-\delta )\tau \rho -\varepsilon^{2}{\left(\gamma -1\right)}^{2}][4(\delta +3)\tau \rho -\alpha^{2}],$$

(13) $$N=6(\delta +3)[2\tau \rho (1-\delta )-\varepsilon^{2}{\left(\gamma -1\right)}^{2}](1-\delta )[2(\delta +3)\rho \tau -\alpha^{2}]+\alpha^{2}\varepsilon^{4}{\left(\gamma -1\right)}^{4}(\delta +3)+2\tau \rho {\left(\delta -1\right)}^{2}\alpha^{4}.$$

The proof of Proposition A4 is shown in Appendix B.

### 3.3. Model Comparison and Analysis

We obtain the following three corollaries by comparing the optimal solutions of three different scenarios, and the proofs of these corollaries are shown in Appendix B.

**Corollary** **1.** $\varsigma^{C*}=\varsigma^{cc*}>\varsigma^{tr*}$, $p_{l}^{cc*}<p_{l}^{tr*}$, $d^{C*}=d^{cc*}>d^{tr*}$, $\pi_{sc}^{C*}=\pi_{sc}^{cc*}>\pi_{sc}^{tr*}$, $\pi_{sc}^{C*}/d^{C*}=\pi_{sc}^{cc*}/d^{cc*}<\pi_{sc}^{tr*}/d^{tr*}$.

The optimal logistics service level in the contractual cooperation model (centralized model) is higher than that of the decentralized model, then the cooperation of the TPLSP and retailer will bring a higher freshness of fresh agricultural products, which is more healthier to consumers. Therefore, the total demand in the contractual cooperation model (centralized model) is higher than that in the decentralized model. While the TPLSP’s logistics service price in the contractual cooperation model is less than that in the decentralized model. The profit of FASC in the contractual cooperation model (centralized model) is higher than that in the decentralized model. While the marginal profit of FASC in the contractual cooperation model (centralized model) is less than that in decentralized model.

**Corollary** **2.** $pdl^{C*}=pdl^{cc*}<pdl^{tr*}$ *and* $\left\{ \begin{array}{l} ppl^{C*}=ppl^{cc*}>ppl^{tr*}\begin{array}{cc} & and\begin{array}{cc} & tdl^{C*}=tdl^{cc*}>tdl^{tr*}\begin{array}{cc} \begin{array}{cc} , & \end{array} & mc>mc_{1} \end{array} \end{array} \end{array}\\ ppl^{C*}=ppl^{cc*}<ppl^{tr*}\begin{array}{cc} & and\begin{array}{cc} & tdl^{C*}=tdl^{cc*}>tdl^{tr*}\begin{array}{cc} \begin{array}{cc} , & \end{array} & mc_{2}<mc<mc_{1} \end{array} \end{array} \end{array}\\ ppl^{C*}=ppl^{cc*}<ppl^{tr*}\begin{array}{cc} & and\begin{array}{cc} & tdl^{C*}=tdl^{cc*}<tdl^{tr*}\begin{array}{cc} \begin{array}{cc} , & \end{array} & mc_{3}<mc<mc_{2} \end{array} \end{array} \end{array} \end{array}\right.$ *where* $mc=c_{l}+\omega =(1-\beta )/\rho -mc_{sm}$ *which can be interpreted as the smallest marginal cost of FASC,*

(14) $$mc_{1}=\frac{1}{\rho }-\frac{\left\{(\delta +3)[2\tau \rho (1-\delta )-{\left(\gamma -1\right)}^{2}\varepsilon^{2}]-\alpha^{2}(1-\delta )\}\{4\tau^{2}\rho^{2}(1-\delta )(\delta +3)+{\left(\gamma -1\right)}^{2}\varepsilon^{2}\alpha^{2}\right\}}{2(1-\delta )\alpha \rho \{[2\tau (\delta +3)\rho +\alpha^{2}]{\left(\gamma -1\right)}^{2}\varepsilon^{2}+8\tau^{2}\rho^{2}(1-\delta )(\delta +3)\}}-mc_{sm},$$

(15) $$\begin{array}{l} mc_{2}=\frac{1}{\rho }-mc_{sm}\\ +\frac{-\{-[2(\delta +3)\tau \rho -\alpha^{2}]{\left(\gamma -1\right)}^{2}\varepsilon^{2}+4\tau^{2}\rho^{2}(1-\delta )(\delta +3)]\}\{(\delta +3)[2\tau \rho (1-\delta )-{\left(\gamma -1\right)}^{2}\varepsilon^{2}]-\alpha^{2}(1-\delta )\}\{4\tau \rho (\delta +3)-\alpha^{2}\}}{2\alpha \rho \{24{\left(1-\delta \right)}^{2}{\left(3+\delta \right)}^{2}\tau^{3}\rho^{3}-8[{\left(\gamma -1\right)}^{2}(\delta +3)\varepsilon^{2}+(1-\delta )\alpha^{2}](\delta +3)(1-\delta )\tau^{2}\rho^{2}-2{\left(\gamma -1\right)}^{2}(\delta +3)\varepsilon^{2}\tau \rho [2(\delta -1)\alpha^{2}+{\left(\gamma -1\right)}^{2}(\delta +3)\varepsilon^{2}]+\alpha^{4}{\left(\gamma -1\right)}^{2}\varepsilon^{2}(\delta -1)\}} \end{array},$$

*And*

(16) $$mc_{3}=\frac{1}{\rho }-\frac{\left(\delta +3)[2\tau \rho (1-\delta )-{\left(\gamma -1\right)}^{2}\varepsilon^{2}]-\alpha^{2}(1-\delta \right)}{2(1-\delta )\alpha \rho }-mc_{sm}.$$

From an environmental perspective, the per unit demand volume loss in the contractual cooperation model (centralized model) is always less than that in the decentralized model. While the relationships between the other two types of volume losses in the contractual cooperation model (centralized model) and those in the decentralized model are related to the smallest marginal cost of FASC. The dividing points of the smallest marginal cost of FASC are related to the consumer utility discount coefficient, the service sensitivity coefficient, the natural volume loss rate, the freshness level discount coefficient, the service cost factor, the spot price in the spot market, and the price sensitivity coefficient. The per unit profit volume loss and total demand volume loss in the contractual cooperation model (centralized model) can be lower than those in the decentralized model only when the smallest marginal cost of FASC is low. As the smallest marginal cost of FASC is always more than 0, the per unit profit volume loss and total demand volume loss in the contractual cooperation model will always be higher than those in the decentralized model when $mc_{1}\leq 0$ and the total demand volume loss in the contractual cooperation model will always be higher than that in the decentralized model when $mc_{2}\leq 0$.

**Corollary** **3.** *If*${\left(1-\delta \right)}^{2}\tau \rho >2{\left(\gamma -1\right)}^{2}\varepsilon^{2}$*, The Hessian matrix of smallest marginal cost of FASC denoted as*$mc_{3}$*is inversely proportional to the consumer utility discount coefficient otherwise,*$mc_{3}$*is directly proportional to it.*$mc_{3}$*is directly proportional to the service sensitivity coefficient and natural volume loss rate. If*$(\delta +3)[2\tau \rho (1-\delta )-2\alpha (1-\gamma )\varepsilon -{\left(\gamma -1\right)}^{2}\varepsilon^{2}]+\alpha^{2}(1-\delta )<0$, $mc_{3}$*is inversely proportional to the freshness level discount coefficient otherwise,*$mc_{3}$*is directly proportional to it. If*$2mc_{sm}\rho \{(\delta +3)[2(1-\delta )\rho \tau -{\left(1-\gamma \right)}^{2}\varepsilon^{2}]-(1-\delta )\alpha^{2}\}-(1-\delta )\alpha^{2}[(1+\gamma )\varepsilon +2(1-mc_{sm}\rho )]-\alpha {\left(1-\gamma \right)}^{2}\varepsilon^{2}(\delta +3)<0$, $mc_{3}$*is inversely proportional to the price sensitivity coefficient otherwise,*$mc_{3}$*is directly proportional to it.*

The following results are shown from Corollary 3 under the conditions of *L_i_* (*i = A,B*): (1) If 0<δ<G(γ) where $G(\gamma )=1-\sqrt{2/\left(\tau \rho \right)}(1-\gamma )\varepsilon$, then $mc_{3}$ decreases with *δ* and if 1>δ>G(γ), then $mc_{3}$ increases with *δ*. (2) if $2{\left(c_{sm}+\varepsilon \right)}^{2}+\tau \rho (\delta -1)>0$ and 0<γ<H(δ) where $H(\delta )=\left\{-(2\varepsilon +4mc_{sm})+\sqrt{\left(\delta +3)[4{\left(c_{sm}+\varepsilon \right)}^{2}+2\tau \rho (\delta -1)\right]}\right\}/2\varepsilon$, then $mc_{3}$ decreases with *γ* and if $2{\left(c_{sm}+\varepsilon \right)}^{2}+\tau \rho (\delta -1)>0$ and 1>γ>H(δ), then $mc_{3}$ increases with *γ*, and if $2{\left(c_{sm}+\varepsilon \right)}^{2}+\tau \rho (\delta -1)<0$, then $mc_{3}$ increases with *γ*.

## 4. Numerical Experimentation

According to the above assumptions and preconditions that each parameters should meet in the models, the specific values of them are set at *δ* = 0.5, *ε* = 0.7, *γ* = 0.5, *τ* = 0.9, *ρ* = 0.9, *ω* = 0.2, *m* = 0.2, *c_sm_* = 0.8, and *c_l_* = 0.1. The results are detailed in Table 2.

In this numerical experimentation, we have $mc_{1}>mc>mc_{2}$. It can be verified from Table 2 that the profit of FASC, the optimal logistics service level, and the total demand in the centralized model are higher than those in the decentralized model. Due to $mc_{1}>mc>mc_{2}$, both the per unit demand volume loss and per unit profit volume loss in the centralized model (contractual cooperation model) are lower than those in the decentralized model, while the total demand volume loss in the centralized model (contractual cooperation model) is higher than that in the decentralized model. Next, we discuss the impact of parameters (*δ*, *ε*, *γ*, *ρ*) on the profit of FASC and three types of volume losses in the centralized model and decentralized model.

### 4.1. The Impact of δ on the Profit of FASC and Three Types of Volume Losses

**Figure 3:**
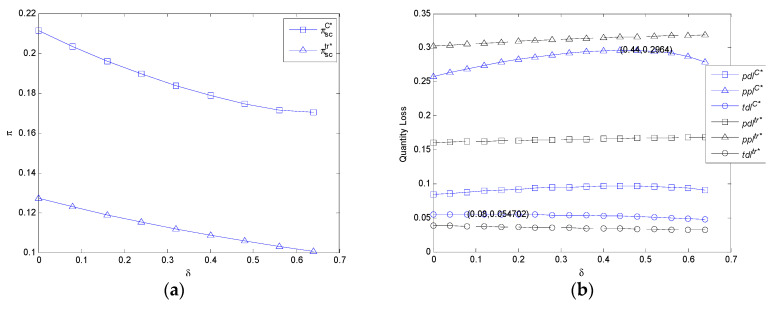
The impact of δ on the profit of FASC and three types of volume losses in the centralized model and decentralized model. (**a**) The impact of *δ* on the profit of FASC in the centralized model and decentralized model and (**b**) the impact of *δ* on three types of volume losses in the centralized model and decentralized model.

It can be observed from Figure 3a that the profit of FASC in two models decreases with the consumer utility discount coefficient. With the increase of the consumer utility discount coefficient, the profit of FASC in the centralized model is always higher than that of the decentralized model.

**Figure 4:**
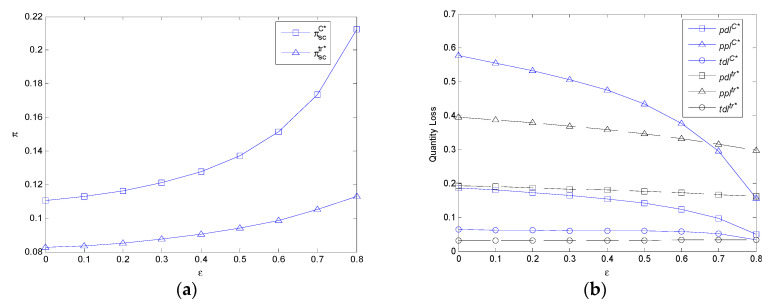
The impact of *ε* on profit of FASC and three types of volume losses in the centralized model and decentralized model. (**a**) The impact of *ε* on the profit of FASC in the centralized model and decentralized model. (**b**) The impact of *ε* on three types of volume losses in the centralized model and decentralized model.

It can be observed from Figure 3b that the per unit demand volume loss, the per unit profit volume loss, and the total demand volume loss in the centralized model increases first and then decreases with the consumer utility discount coefficient. Additionally, when *δ* = 0.44, the per unit demand volume loss and per unit profit volume loss in the centralized model both reach the maximum at the same time, while the total demand volume loss in the centralized model reaches the maximum when *δ* = 0.08 as shown in Figure 4. The per unit demand volume loss and per unit profit volume loss in the decentralized model increases with the consumer utility discount coefficient, while the total demand volume loss in the decentralized model decreases with the consumer utility discount coefficient. With the increase of the consumer utility discount coefficient, both the per unit demand volume loss and per unit profit volume loss in the centralized model are lower than those in the decentralized model because of $mc_{1}>mc>mc_{2}$, while the total demand volume loss in the centralized model is higher than that of the decentralized model because of $mc_{1}>mc>mc_{2}$.

### 4.2. The Impact of ε on the Profit of FASC and Three Types of Vol. The Impact of γ on the Profit of FASC and Three Types of Volume Losses ume Losses

It can be observed from Figure 4a that the profit of FASC in the two models increases with service sensitivity. With the increase of service sensitivity, the profit of FASC in the centralized model is always higher than that of the decentralized model.

It can be observed from Figure 4b that the per unit demand volume loss, the per unit profit volume loss, and the total demand volume loss in the centralized model decrease with service sensitivity. Additionally, the per unit demand volume loss and per unit profit volume loss in the decentralized model decreases with service sensitivity. While the total demand volume loss in the decentralized model decreases first and then increases with service sensitivity. The per unit demand volume loss in the decentralized model is always higher than that of the centralized model with the increase of service sensitivity. In addition, the per unit profit volume loss in the centralized model is lower than that of the decentralized model because of $mc_{1}>mc>mc_{2}$ when *ε >* 0.674, while the total demand volume loss in the centralized model is lower than that of the decentralized model because of $mc_{3}<mc<mc_{2}$ when *ε >* 0.8.

### 4.3. The Impact of γ on the Profit of FASC and Three Types of Volume Losses

**Figure 5:**
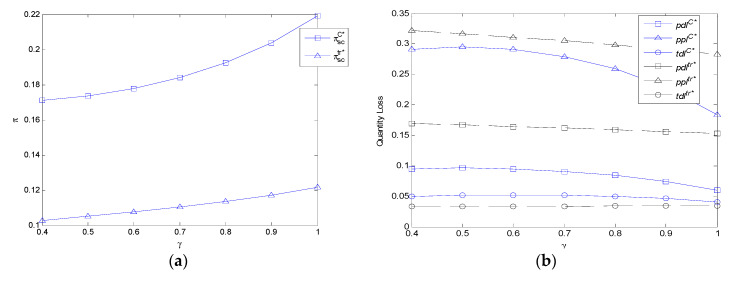
The impact of *γ* on profit of FASC and three types of volume losses in the centralized model and decentralized model. (**a**) The impact of *γ* on the profit of FASC in the centralized model and decentralized model. (**b**) The impact of *γ* on three types of volume losses in the centralized model and decentralized model.

It can be observed from Figure 5a that the profit of FASC in the two models increases with the freshness level discount coefficient. With the increase of the freshness level discount coefficient, the profit of FASC in the centralized model is always higher than that of the decentralized model.

It can be observed from Figure 5b that the per unit demand volume loss and per unit profit volume loss in the centralized model increase first and then decrease with the freshness level discount coefficient because of $2{\left(c_{sm}+\varepsilon \right)}^{2}+\tau (\delta -1)>0$ in this numerical experimentation. Additionally, the total demand volume loss in the centralized model also increases first and then decreases with the freshness level discount coefficient in this numerical experimentation. While the per unit demand volume loss, per unit profit volume loss, and total demand volume loss in the decentralized model decrease with the freshness level discount coefficient. With the increase of the freshness level discount coefficient, the per unit demand volume loss and per unit profit volume loss in the centralized model are lower than those in the decentralized model because of $mc_{1}>mc>mc_{2}$. The total demand volume loss in the centralized model is higher than that of the decentralized model because of $mc_{1}>mc>mc_{2}$ when the freshness level discount coefficient is small, and it is lower than that of the decentralized model because of $mc_{3}<mc<mc_{2}$ when the freshness level discount coefficient is high.

### 4.4. The Impact of ρ on the Profit of FASC and Three Types of Volume Losses

**Figure 6:**
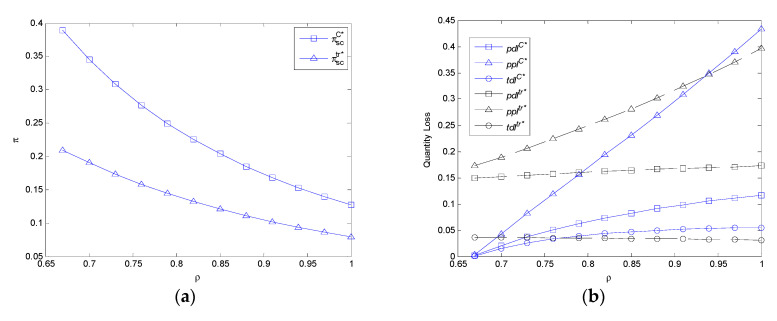
The impact of *ρ* on profit of FASC and three types of volume losses in the centralized model and decentralized model. (**a**) The impact of *ρ* on profit of FASC in the centralized model and decentralized model. (**b**) The impact of *ρ* on three types of volume losses in the centralized model and decentralized model.

It can be observed from Figure 6a that the profit of FASC in two models decreases with the price sensitivity coefficient. With the increase of price sensitivity, the profit of FASC in the centralized model is always higher than that of the decentralized model.

It can be observed from Figure 6b that the per unit demand volume loss, per unit profit volume loss, and total demand volume loss in the centralized model increase with the price sensitivity coefficient in this numerical experimentation. In addition, the per unit demand volume loss and per unit profit volume loss in the decentralized model increase with it. While the total demand volume loss in the decentralized model decreases with it. With the increase of the price sensitivity coefficient, the per unit demand volume loss in the centralized model is lower than that in decentralized model. Furthermore, the per unit profit volume loss in the centralized model is lower than that in the decentralized model because of $mc_{1}>mc>mc_{2}$ when *ρ <* 0.94, while the total demand volume loss in the centralized model is lower than that in the decentralized model because of $mc_{3}<mc<mc_{2}$ when *ρ <* 0.76.

## 5. Discussion

Based on consumer strategic behavior, the utility functions of two periods during retail are considered respectively. Then, the demand function is derived from the utility function and profit models are established to investigate the two-stage pricing, coordination, and volume loss reduction of the supply chain, considering the characteristics of fresh agricultural products including the physical volume loss during transport and quality loss during retail in the presence of strategic consumers. In general, we obtained the following results in this paper:

(1) The fresh agricultural products supply chain where the TPLSP and retailer act as a Stackelberg leader and a follower can achieve coordination by the revenue and service-cost sharing contract. This is compatible with the study of Ma et al. [10] however the unit price for the freshness-keeping service must be negative in order to achieve coordination. Differing from it, the unit price of logistics service can be positive in this paper and when the revenue sharing coefficient and service-cost sharing coefficient are under certain conditions, the Pareto improvement can be achieved. The cost sharing coefficient (1−μ) decreases with the consumer utility discount coefficient. Therefore the consumers’ strategic behavior influences the profit distribution which is compatible with the study of Su et al. [26]. The cooperation of the TPLSP and retailer will bring logistics service level, which leads to higher freshness. Then fresh agricultural products are healthier to consumers. Therefore, retailers and TPLSP should try their best to cooperate and negotiate the profit sharing coefficient to obtain increased profit and higher freshness of fresh agricultural products;
(2) The supply chain coordination leads to a reduction in the per unit demand volume loss which is compatible with the study of Mohammadi et al. [6]. The per unit demand volume loss decreases with the optimal logistics service level [57] and the optimal logistics service level is higher in contractual cooperation model than in a decentralized model because of the retailer’s revenue and service-cost sharing with the TPLSP. Then the per unit demand volume loss is always less compared to the decentralized model. Obliviously, the corporation of the retailer and TPLSP will get more profit and a reduction in the per unit demand volume loss [53];
(3) The supply chain coordination leads to a reduction in the per unit profit volume loss and total demand volume loss simultaneously only if the lowest marginal costs of FASC occur under certain conditions. As the study of Bai et al. [24] shows the total carbon emission are analyzed, the two types of volume losses are not studied in the model studies to the best of the author’s knowledge. However the two types of volume losses are important for decision makers. In the contractual cooperation model, the freshness of products are higher and the total demands are higher than in the decentralized model. Therefore the two types of volume losses are not always lower than in the decentralized model. The two types of volume losses can be simultaneously lower than in the decentralized model only if the lowest marginal costs is low. Then the retailer and TPLSP should reduce the smallest marginal cost of the supply chain, so that the two types of volume losses are reduced. In addition, the increase of the lower bound of the smallest marginal cost of FASC will increase the possibility that the coordination leads to a reduction in the two types of volume losses. According to the relationships between the lower bound of the smallest marginal cost of FASC and parameters in the model, the increase in the service sensitivity coefficient, the decrease (increase) in consumer utility discount coefficient if it is small (large), and the increase in the freshness discount coefficient if it is large will lead to the increase in the possibility of a reduction in the two types of volume losses when the supply chain achieves coordination. Therefore, while it seemed that volume loss was not related to the retailer, it in fact has a lot to do with the retailer because of their decisions about the freshness discount coefficient and influences on consumers to change their strategy, thus these two participants of the supply chain cannot work separately but rather make a collaborative effort to reduce volume losses [53].
(4) When the FASC achieves coordination the profit of FASC is inversely proportional to the consumer utility discount coefficient which is compatible with the study of Chen et al. [17], making the decision of responding pricing. The total demand is inversely proportional to the consumer utility discount coefficient. Therefore, consumer strategic behavior is detrimental to the total demand which is also compatible with the study of Chen et al. [17]. The optimal logistics service level decreases first and then increases with the consumer utility discount coefficient. If the level of strategic behavior is small (large), the decrease (increase) in it will lead to higher freshness of products and less per unit demand volume loss. Therefore, the increase in the level of strategic behavior is detrimental to the profits of the whole supply chain, but beneficial to consumers and the environment under certain conditions. As mentioned above, the level of strategic behavior also influences the profit distribution between the retailer and TPLSP, then the increase in it will change the cost sharing which may be beneficial to the retailer;
(5) When FASC achieves coordination, the profit of FASC is inversely proportional to the price sensitivity coefficient, and it is directly proportional to the service sensitivity coefficient and freshness level discount coefficient due to the utility of consumers being inversely proportional (directly proportional) to the price sensitivity coefficient (service sensitivity coefficient). The lower (higher) of the price sensitivity coefficient (service sensitivity coefficient), the more total demand. The relationships between the profit and price sensitivity (freshness sensitivity) is compatible with the study of Yan et al. [7]. In the numerical analysis of Yan et al. [7], it seems that the profit decreases first and then increases with price sensitivity (freshness sensitivity) due to the neglect of the condition that the order quantity must be positive. When this condition is taken into account, the relationship between the profit of the supply chain and the price sensitivity (freshness sensitivity) is the same as this paper;
(6) When FASC achieves coordination, the relationships between the three types of volume losses including the per unit demand volume loss, per unit profit volume loss, and total demand volume loss in the centralized model (contractual cooperation model) and parameters are as follows: (1) The three types of volume losses are inversely proportional to the service sensitivity coefficient which is compatible with the study of Mohammadi et al. [6] where the percentage of product waste is inversely proportional to surviving rate coefficient therefore, the TPLSP should arrange the storage of products in transport vehicles reasonably and load fresh agricultural products into transport vehicles as soon as possible so as to reduce loss and increase the freshness of the products and profit of FASC; (2) the three types of volume losses increase first and then decrease with the consumer utility discount coefficient due to the relationship between the logistics service and consumer utility discount coefficient. Therefore, the retailer should emphasize the nutritional value of fresh agricultural products to consumers in order to affect them to reduce their consumer utility discount coefficient when it’s small so as to reduce loss and increase the profit of FASC. It should be noted that the impact of the consumer utility discount coefficient on profit distribution is not considered here; (3) when $2{\left(c_{sm}+\varepsilon \right)}^{2}+\tau \rho (\delta -1)>0$, the per unit demand volume loss and per unit profit volume loss first increase and then decrease with the freshness discount coefficient; when $2{\left(c_{sm}+\varepsilon \right)}^{2}+\tau \rho (\delta -1)<0$, the two types of volume losses decrease with it. Therefore, the retailer should increase the freshness level discount coefficient if it is large enough to reduce the two types of losses and increase the profit of FASC; (4) the per unit demand volume loss and per unit profit volume loss increase with the price sensitivity coefficient. Therefore, the retailer should emphasize the nutritional value of fresh agricultural products to consumers in order to affect them to reduce their price sensitivity coefficient so as to reduce loss and increase the profit of FASC.

## 6. Conclusions

Fresh agricultural product loss is an important problem due to its impacts on society, the economy, and environment. The cooperation or coordination of the whole supply chain will reduce food loss and waste. In this paper, three models (centralized model, decentralized model, and contractual cooperation model) for fresh agricultural products supply chain where the TPLSP and the retailer were risk-neutral and acted as a Stackelberg leader and follower were investigated. The physical volume loss during transport and quality loss during retail in the presence of strategic consumers were taken into account due to the perishable nature of the products and retailers’ dynamic pricing strategy in practice. The two-stage pricing decision and coordination were made by using the equilibrium theory, Stackelberg Game, and backward induction method. The volume reduction were analyzed through a comparison and the impacts of the parameters on profit and the three types volume loss of the supply chain were analyzed. The following conclusions were drawn: (1) FASC achieved coordination by revenue and service-cost sharing contract; (2) the coordination led to a reduction in the per unit demand volume, the coordination led to a reduction in the per profit volume loss and the total demand volume loss simultaneously only if the lowest marginal costs of FASC occurred under certain conditions; and (3) when FASC achieved coordination the increase in the service sensitivity coefficient, the increase in the freshness discount coefficient under certain conditions, the decrease in the consumer benefit discount coefficient under certain conditions, and the decrease in the price sensitivity coefficient led to an increase in the profit of FASC and a reduction in the three types of volume losses.

In this paper, all consumers were assumed to be strategic. Therefore, there is a need to conduct further research on the market where there are both strategic and myopic consumers. Then the further research will be more in line with the actual situation. In addition, in order to facilitate analysis, the volume loss during retail was not taken into account. Therefore, further research on the physical volume loss along the whole supply chain is needed. Furthermore, in the model, the maximization of the profit was taken as the goal and then volume loss was analyzed after the supply chain achieved coordination. Therefore, the coordination led to an increase in the volume loss under some conditions, the purpose of volume loss reduction could not be achieved. Then the purpose of volume loss reduction may be achieved if the minimization of the volume loss was also taken as the goal. This direction may be taken into account in future research to reduce volume losses.

## Figures and Tables

**Table 1:** Notations.

Notation	Description
*u_i_*	The utility functions of consumers in period *i* (*i* = 1, 2)
*v*	The consumers’ basic cognitive value of fresh agricultural products following a uniform distribution of [0,1]
*p_i_*	The selling price in period *i* (*i* = 1, 2)
ς	Logistics service level
*ε*	The measure of the sensitivity of the utility of consumers to the logistics service level, referred to as service sensitivity coefficient
*ρ*	The measures of the sensitivity of the utility of consumers to the selling price, referred to as price sensitivity coefficient
*k*	The measures of the sensitivity of the utility of consumers to the freshness level
*h*	The measures of the sensitivity of freshness level in period one to the logistics service level
(*d_i_, d*)	The demand of the retailer in period *i*, the total demand of the retailer
*c_l_*	Unit cost of the third-party logistics service providers (TPLSP)
*ω*	Unit purchase price of retailer
*p_l_*	The TPLSP’s logistics service price
*δ*	The consumer utility discount coefficient in period 2 within (0,1)
*γ*	Freshness level discount coefficient in period 2 within (0,1)
*π_i_*	The profit of TPSP (*i = l*), retailer (*i = r*), whole supply chain (*i = sc*)
*c_sm_*	The spot price in the spot market
*τ*	The service cost factor
*m*	The loss rate of quantity without logistics service, i.e., natural volume loss rate, within (0,1)
(*pdl*, *ppl*, *tdl*)	The per unit demand volume loss, per unit profit volume loss and total demand volume loss

**Table 2:** The optimal price, demand, profit, and three types of volume loss under different models.

Models	Centralized Model	Decentralized Model
The optimal selling price in the first period	0.969	1.041
The optimal selling price in the second period	0.720	0.954
The optimal logistics service level	0.519	0.164
The optimal logistics service price	-	0.487
The total demand	0.533	0.199
The profit of retailer	-	0.040
The profit of third-party logistics service provider	-	0.065
The profit of supply chain	0.174	0.105
The per unit demand volume loss	0.096	0.167
The per unit profit volume loss	0.296	0.317
The total demand volume loss	0.051	0.033

## Appendix A

### Decentralized Model

Since the retailer purchases all the demand in advance and outsources it to a TPLSP for transport, and the volume loss of the product during transport is obtained from the spot market, the profit of the retailer and the profit of TPLSP are respectively:

(A1) $$\pi_{r}^{tr}(p_{1}^{tr},p_{2}^{tr})=(p_{1}^{tr}-\omega )d_{1}^{tr}+(p_{2}^{tr}-\omega )d_{2}^{tr}-p_{l}^{tr}(d_{1}^{tr}+d_{2}^{tr})-c_{sm}(d_{1}^{tr}+d_{2}^{tr})(1-\varsigma^{tr})m,$$

(A2) $$\pi_{l}^{tr}(p_{l}^{tr},\varsigma^{tr})=(p_{l}^{tr}-c_{l})(d_{1}^{tr}+d_{2}^{tr})-\tau {\left(\varsigma^{tr}\right)}^{2}/2.$$

In this game, the TPLSP and retailer act as a Stackelberg leader and a follower respectively, they aim to maximize their own profit. So the decentralized model is a bilevel programming problem which can be stated as:

(A3) $$\left\{ \begin{array}{l} \left(p_{l}^{tr*},\varsigma^{tr*})\in \arg\max\pi_{l}^{tr}(p_{l}^{tr},\varsigma^{tr},p_{1}^{tr}(p_{l}^{tr},\varsigma^{tr}),p_{2}^{tr}(p_{l}^{tr},\varsigma^{tr})\right)\\ s.t.\begin{array}{cc} & \left(p_{1}^{tr}(p_{l}^{tr},\varsigma^{tr}),p_{2}^{tr}(p_{l}^{tr},\varsigma^{tr}))\in \arg\max\pi_{r}^{tr}(p_{1}^{tr},p_{2}^{tr}\right) \end{array} \end{array}\right..$$

According to the above game order, we can obtain the following Proposition A1.

**Proposition** **A1.**
*Under the conditions of L_j_ (j = C,D), the optimal price in two periods, the optimal logistics service level and the optimal TPLSP’s logistics service price in the decentralized model where the TPLSP act as a Stackelberg leader are:*

(A4) $$\left\{ \begin{array}{l} p_{1}^{tr*}=\frac{1}{\rho }-\frac{\beta [2\tau \rho (1+\delta )-\alpha \varepsilon (1+\gamma )]}{[4\tau \rho (3+\delta )-\alpha^{2}]\rho }\\ p_{2}^{tr*}=\frac{1}{\rho }-\frac{2\beta (2\tau \rho -\alpha \varepsilon \gamma )}{[4\tau \rho (3+\delta )-\alpha^{2}]\rho }\\ \varsigma^{tr*}=\frac{2\alpha \beta }{4\tau \rho (3+\delta )-\alpha^{2}}\\ p_{l}^{tr*}=c_{l}+\frac{2\tau \rho \beta (\delta +3)}{4\tau \rho (3+\delta )-\alpha^{2}} \end{array}\right..$$

**Proof of Proposition** **A1.** The Hessian matrix of $\pi_{r}^{tr}(p_{1}^{tr},p_{2}^{tr})$ about $\left(p_{1}^{tr},p_{2}^{tr}\right)$ is $\left|\begin{array}{cc} \frac{\partial^{2}\pi_{r}^{tr}}{\partial {\left(p_{{}_{1}}^{tr}\right)}^{2}} & \frac{\partial^{2}\pi_{r}^{tr}}{\partial p_{1}^{tr}\partial p_{2}^{tr}}\\ \frac{\partial^{2}\pi_{r}^{tr}}{\partial p_{2}^{tr}\partial p_{1}^{tr}} & \frac{\partial^{2}\pi_{r}^{tr}}{\partial {\left(p_{2}^{tr}\right)}^{2}} \end{array}\right|=\left|\begin{array}{cc} \frac{2\rho }{\delta -1} & \rho \frac{\delta +1}{1-\delta }\\ \rho \frac{\delta +1}{1-\delta } & \frac{2\rho }{\delta -1} \end{array}\right|=\rho^{2}\frac{\delta +3}{1-\delta }>0$, we can get $\frac{\partial^{2}\pi_{r}^{tr}}{\partial {\left(p_{{}_{1}}^{tr}\right)}^{2}}=\frac{\partial^{2}\pi_{r}^{tr}}{\partial {\left(p_{{}_{2}}^{tr}\right)}^{2}}<0$, so $\pi_{r}^{tr}(p_{1}^{tr},p_{2}^{tr})$ is a strictly differentiable concave function of $\left(p_{1}^{tr},p_{2}^{tr}\right)$. The optimal prices in two periods are arrived at after: solving the first-order condition $\left\{ \begin{array}{l} \frac{\partial \pi_{r}^{tr}}{\partial p_{1}^{tr}}=0\\ \frac{\partial \pi_{r}^{tr}}{\partial p_{2}^{tr}}=0 \end{array}\right.$, then we can obtain:

(A5) $$\left\{ \begin{array}{l} p_{1}^{tr}(p_{l}^{tr},\varsigma^{tr})=\frac{\left[(1-\varsigma^{tr})mc_{sm}+p_{l}^{tr}+\omega ]\rho (1+\delta )+2+\varepsilon (1+\gamma \right)}{(\delta +3)\rho }\\ p_{2}^{tr}(p_{l}^{tr},\varsigma^{tr})=\frac{[(\delta \gamma +2\gamma -1)\varepsilon ]\varsigma^{tr}+2[(1-\varsigma^{tr})mc_{sm}+p_{l}^{tr}+\omega ]\rho +1+\delta }{(\delta +3)\rho } \end{array}\right..$$

Substitute Equation (A5) into the profit function of TPLSP, we get:

(A6) $$\pi_{l}^{tr}(p_{l}^{tr},\varsigma^{tr})=\frac{-\tau \varsigma^{tr}(\delta +3)-4[\frac{(\gamma +1)\varepsilon }{2}+\rho mc_{sm}](c_{l}-p_{l}^{tr})\varsigma^{tr}+4(c_{l}-p_{l}^{tr})[(mc_{sm}+p_{l}^{tr}+\omega )\rho -1]}{2\delta +6}.$$

The Hessian matrix of $\pi_{l}^{tr}(p_{l}^{tr},\varsigma^{tr})$ about $\left(p_{l}^{tr},\varsigma^{tr}\right)$ is $\left|\begin{array}{cc} \frac{\partial^{2}\pi_{l}^{tr}}{\partial {\left(p_{{}_{l}}^{tr}\right)}^{2}} & \frac{\partial^{2}\pi_{l}^{tr}}{\partial p_{l}^{tr}\partial \varsigma^{tr}}\\ \frac{\partial^{2}\pi_{l}^{tr}}{\partial \varsigma^{tr}\partial p_{l}^{tr}} & \frac{\partial^{2}\pi_{l}^{tr}}{\partial {\left(\varsigma^{tr}\right)}^{2}} \end{array}\right|=\left|\begin{array}{cc} \frac{-4\rho }{\delta +3} & \frac{2\rho mc_{sm}+(1+\gamma )\varepsilon }{\delta +3}\\ \frac{2\rho mc_{sm}+(1+\gamma )\varepsilon }{\delta +3} & -\tau \end{array}\right|=\frac{4\rho \tau (\delta +3)-\alpha^{2}}{{\left(\delta +3\right)}^{2}}$. Under the condition of LC, we can obtain $\left|\begin{array}{cc} \frac{\partial^{2}\pi_{l}^{tr}}{\partial {\left(p_{{}_{l}}^{tr}\right)}^{2}} & \frac{\partial^{2}\pi_{l}^{tr}}{\partial p_{l}^{tr}\partial \varsigma^{tr}}\\ \frac{\partial^{2}\pi_{l}^{tr}}{\partial \varsigma^{tr}\partial p_{l}^{tr}} & \frac{\partial^{2}\pi_{l}^{tr}}{\partial {\left(\varsigma^{tr}\right)}^{2}} \end{array}\right|>0$. Since $\frac{\partial^{2}\pi_{l}^{tr}}{\partial {\left(p_{{}_{l}}^{tr}\right)}^{2}}<0$ and $\frac{\partial^{2}\pi_{l}^{tr}}{\partial {\left(\varsigma^{tr}\right)}^{2}}<0$, we can obtain $\pi_{l}^{tr}(p_{l}^{tr},\varsigma^{tr})$ is a strictly differentiable concave function of $\left(p_{l}^{tr},\varsigma^{tr}\right)$. The optimal logistics service level and the optimal TPLSP’s logistics service price are arrived at after solving the first-order condition $\left\{ \begin{array}{l} \frac{\partial \pi_{l}^{tr}}{\partial p_{l}^{tr}}=0\\ \frac{\partial \pi_{l}^{tr}}{\partial \varsigma^{tr}}=0 \end{array}\right.$, then we can obtain:

(A7) $$\left\{ \begin{array}{l} \varsigma^{tr*}=\frac{2\alpha \beta }{4\rho \tau (3+\delta )-\alpha^{2}}\\ p_{l}^{tr*}=c_{l}+\frac{2\tau \beta (\delta +3)}{4\rho \tau (3+\delta )-\alpha^{2}} \end{array}\right..$$

Substitute Equation (A7) into Equation (A5), we can obtain:

(A8) $$\left\{ \begin{array}{l} p_{1}^{tr*}=\frac{1}{\rho }-\frac{\beta [2\rho \tau (1+\delta )-\alpha \varepsilon (1+\gamma )]}{[4\tau \rho (3+\delta )-\alpha^{2}]\rho }\\ p_{2}^{tr*}=\frac{1}{\rho }-\frac{2\beta (2\rho \tau -\alpha \varepsilon \gamma )}{[4\tau \rho (3+\delta )-\alpha^{2}]\rho } \end{array}\right..$$

We can get $p_{1}^{tr*}>p_{2}^{tr*}$. Substitute Equation (A7) and Equation (A8) into the demand function, we can get $d_{2}^{tr*}=\frac{\beta [2\rho \tau (1-\delta )+(\gamma -1)\alpha \varepsilon ]}{\left[4(\delta +3)\rho \tau -\alpha^{2}](1-\delta \right)}>0$. under the condition of *L_D_*. And under the condition of *L_C_*, $0<\varsigma^{tr*}<1$ is satisfied. □

Substitute Equation (A4) into the demand function (2) and profit functions (A1) and (A2), we get:

(A9) $$\left\{ \begin{array}{l} d_{1}^{tr*}=\frac{\beta [2\tau \rho (1-\delta )-(\gamma -1)\alpha \varepsilon ]}{\left[4(\delta +3)\tau \rho -\alpha^{2}](1-\delta \right)}\\ d_{2}^{tr*}=\frac{\beta [2\tau \rho (1-\delta )+(\gamma -1)\alpha \varepsilon ]}{\left[4(\delta +3)\tau \rho -\alpha^{2}](1-\delta \right)}\\ d^{tr*}=\frac{4\beta \tau \rho }{4(\delta +3)\tau \rho -\alpha^{2}}\\ \pi_{r}^{tr*}=\frac{4(3-\delta^{2}-2\delta )\tau^{2}\rho^{2}+{\left(\gamma -1\right)}^{2}\varepsilon^{2}\alpha^{2}}{{\left[4(\delta +3)\tau \rho -\alpha^{2}\right]}^{2}(1-\delta )\rho }\beta^{2}\\ \pi_{l}^{tr*}=\frac{2\beta^{2}\tau }{4(\delta +3)\tau \rho -\alpha^{2}}\\ \pi_{sc}^{tr*}=\frac{\beta^{2}[12(3-\delta^{2}-2\delta )\tau^{2}\rho^{2}-2\alpha^{2}(1-\delta )\tau \rho +\alpha^{2}{\left(\gamma -1\right)}^{2}\varepsilon^{2}]}{{\left[4(\delta +3)\tau \rho -\alpha^{2}\right]}^{2}(1-\delta )\rho } \end{array}\right..$$

The following results are shown in the decentralized model with the TPLSP as the leader under the conditions of *L_j_* (*j = C,D*): (1) if $-\alpha^{2}+2\alpha \varepsilon (1+\gamma )+8\tau \rho >0$, the selling price in period one is inversely proportional to the consumer utility discount coefficient; otherwise, it is directly proportional to the consumer utility discount coefficient; (2) the logistics service level, the demand in two periods, the total demand, the TPLSP’s logistics service price, the profit of the TPLSP, the profit of the retailer, and the profit of FASC are inversely proportional to the consumer utility discount coefficient; while the selling price in the second period is directly proportional to it; (3) the selling price in two periods, the optimal logistics service level, the demand in two periods, the total demand, the TPLSP’s logistics service price, the profit of the TPLSP, the profit of the retailer, and the profit of FASC are directly proportional to the service sensitivity coefficient, and they are directly proportional to the freshness level discount coefficient; (4) if $\alpha^{2}-4(\delta +3)\tau \rho +4\alpha \beta >0$, the total demand and the optimal TPLSP’s logistics service price are directly proportional to the natural volume loss rate; otherwise, they are inversely proportional to it; (5) the marginal profit of the TPLSP is β/(2ρ).

## Appendix B

$\alpha =2\rho mc_{sm}+(1+\gamma )\varepsilon$, $\beta =1-(mc_{sm}+c_{l}+\omega )\rho$, *L_A_* is $(\delta +3)[2\rho \tau (1-\delta )-{\left(\gamma -1\right)}^{2}\varepsilon^{2}]-\alpha^{2}(1-\delta )-2(1-\delta )\alpha \beta >0$, *L_B_* is $2\rho \tau (1-\delta )-{\left(\gamma -1\right)}^{2}\varepsilon^{2}+(\gamma -1)\alpha \varepsilon >0$, *L_C_* is $4\rho \tau (3+\delta )-\alpha^{2}-2\alpha \beta >0$, *L_D_* is 2ρτ(1−δ)+(γ−1)αε>0, $mc=(1-\beta )/\rho -mc_{sm}$, $mc_{1}=\frac{1}{\rho }-\frac{\left\{(\delta +3)[2\tau \rho (1-\delta )-{\left(\gamma -1\right)}^{2}\varepsilon^{2}]-\alpha^{2}(1-\delta )\}\{4\tau^{2}\rho^{2}(1-\delta )(\delta +3)+{\left(\gamma -1\right)}^{2}\varepsilon^{2}\alpha^{2}\right\}}{2(1-\delta )\alpha \rho \{[2\tau (\delta +3)\rho +\alpha^{2}]{\left(\gamma -1\right)}^{2}\varepsilon^{2}+8\tau^{2}\rho^{2}(1-\delta )(\delta +3)\}}-mc_{sm}$,$mc_{3}=\frac{1}{\rho }-\frac{\left(\delta +3)[2\tau \rho (1-\delta )-{\left(\gamma -1\right)}^{2}\varepsilon^{2}]-\alpha^{2}(1-\delta \right)}{2(1-\delta )\alpha \rho }-mc_{sm}$.

**Proof of Proposition** **A2.** The profit of FASC is:

(A10) $$\begin{array}{l} \pi_{sc}^{C}(p_{1}^{C},p_{2}^{C},\varsigma^{C})=\frac{\left(p_{1}^{C}-\omega )[1-\rho p_{1}^{C}+\varepsilon \varsigma^{C}-(\varsigma^{C}\gamma \varepsilon -\rho p_{2}^{C}+1)\delta \right]}{1-\delta }+\frac{\left(p_{2}^{C}-\omega )(\rho p_{1}^{C}-\rho p_{2}^{C}-\varepsilon \varsigma^{C}+\varsigma^{C}\gamma \varepsilon \right)}{1-\delta }\\ -(1-\rho p_{2}^{C}+\varsigma^{C}\gamma \varepsilon )[c_{sm}(1-\varsigma^{C})m+c_{l}]-\tau {\left(\varsigma^{C}\right)}^{2}/2 \end{array}$$

The Hessian matrix of

$$\pi_{sc}^{C}(p_{1}^{C},p_{2}^{C},\varsigma^{C})$$

about $\left(p_{1}^{C},p_{2}^{C},\varsigma^{C}\right)$ is $\left|\begin{array}{ccc} \frac{\partial^{2}\pi_{sc}^{C}}{\partial {\left(p_{1}^{C}\right)}^{2}} & \frac{\partial^{2}\pi_{sc}^{C}}{\partial p_{1}^{C}\partial p_{2}^{C}} & \frac{\partial^{2}\pi_{sc}^{C}}{\partial p_{1}^{C}\partial \varsigma^{C}}\\ \frac{\partial^{2}\pi_{sc}^{C}}{\partial p_{2}^{C}\partial p_{1}^{C}} & \frac{\partial^{2}\pi_{sc}^{C}}{\partial {\left(p_{2}^{C}\right)}^{2}} & \frac{\partial^{2}\pi_{sc}^{C}}{\partial p_{2}^{C}\partial \varsigma^{C}}\\ \frac{\partial^{2}\pi_{sc}^{C}}{\partial \varsigma^{C}\partial p_{1}^{C}} & \frac{\partial^{2}\pi_{sc}^{C}}{\partial \varsigma^{C}\partial p_{2}^{C}} & \frac{\partial^{2}\pi_{sc}^{C}}{\partial {\left(\varsigma^{C}\right)}^{2}} \end{array}\right|=\left|\begin{array}{ccc} \frac{2\rho }{\delta -1} & \rho \frac{\delta +1}{1-\delta } & \frac{\varepsilon (\delta \gamma -1)}{\delta -1}\\ \rho \frac{\delta +1}{1-\delta } & \frac{2\rho }{\delta -1} & \frac{\varepsilon (1-\gamma )}{\delta -1}-\rho mc_{sm}\\ \frac{\varepsilon (\delta \gamma -1)}{\delta -1} & \frac{\varepsilon (1-\gamma )}{\delta -1}-\rho mc_{sm} & 2\gamma \varepsilon mc_{sm}-\tau \end{array}\right|$. So the three order principal subexpressions of the matrix are as follows: $D_{1}=2\rho /\left(\delta -1\right)<0$, $D_{2}=\rho^{2}\left(\delta +3\right)/\left(1-\delta \right)>0$, $D_{3}=\rho \frac{\left(\delta +3)[{\left(\gamma -1\right)}^{2}\varepsilon^{2}-2\tau (1-\delta )\rho ]+\alpha^{2}(1-\delta \right)}{2{\left(\delta -1\right)}^{2}}$. Under the condition of *L_A_*, we can obtain $D_{3}<0$, so $\pi_{sc}^{C}(p_{1}^{C},p_{2}^{C},\varsigma^{C})$ is a strictly differentiable concave function of $\left(p_{1}^{C},p_{2}^{C},\varsigma^{C}\right)$. The optimal prices in two periods and the optimal logistics service level are arrived at after solving the first-order condition $\left\{ \begin{array}{l} \frac{\partial \pi_{sc}^{C}}{\partial p_{1}^{C}}=0\\ \frac{\partial \pi_{sc}^{C}}{\partial p_{2}^{C}}=0\\ \frac{\partial \pi_{sc}^{C}}{\partial \varsigma^{C}}=0 \end{array}\right.$, then we can obtain:

(A11) $$\left\{ \begin{array}{l} p_{1}^{C*}=\frac{1}{\rho }-\frac{\beta [2(1-\delta^{2})\tau \rho -(1-\delta )\alpha (\gamma +1)\varepsilon -{\left(\gamma -1\right)}^{2}\varepsilon^{2}(1+\delta )]}{\{(\delta +3)[2\tau \rho (1-\delta )-{\left(\gamma -1\right)}^{2}\varepsilon^{2}]-\alpha^{2}(1-\delta )\}\rho }\\ p_{2}^{C*}=\frac{1}{\rho }-\frac{2\beta [2\tau \rho (1-\delta )-{\left(\gamma -1\right)}^{2}\varepsilon^{2}-(1-\delta )\alpha \gamma \varepsilon ]}{\{(\delta +3)[2\tau \rho (1-\delta )-{\left(\gamma -1\right)}^{2}\varepsilon^{2}]-\alpha^{2}(1-\delta )\}\rho }\\ \varsigma^{C*}=\frac{2(1-\delta )\alpha \beta }{\left(\delta +3)[2\tau \rho (1-\delta )-{\left(\gamma -1\right)}^{2}\varepsilon^{2}]-\alpha^{2}(1-\delta \right)} \end{array}\right..$$

Substitute Equation (A11) into the demand function in the second period, we can get $d_{2}^{C*}=\frac{\beta [2\tau \rho (1-\delta )-{\left(\gamma -1\right)}^{2}\varepsilon^{2}+(\gamma -1)\alpha \varepsilon ]}{\left(\delta +3)[-{\left(\gamma -1\right)}^{2}\varepsilon^{2}+2\tau \rho (1-\delta )]-\alpha^{2}(1-\delta \right)}>0$ under the condition of *L_B_*. And under the condition of *L_A_*, $0<\varsigma^{C*}<1$ is satisfied. We can get $p_{1}^{C*}>p_{2}^{C*}$ from the Equation (A2). □

**Proof of Proposition** **A3.** From the definition of *pdl* and *ppl*, we get:

(A12) $$\left\{ \begin{array}{l} pdl^{C*}=(1-\varsigma^{C*})m\\ ppl^{C*}=(1-\varsigma^{*})md^{C*}/\pi_{sc}^{C*}=2pdl^{C*}\rho /\beta \end{array}\right.$$

From Equations (A2) and (A3) and the demand and profit functions in the centralized model, we get:

(A13) $$\frac{\pi_{sc}^{C*}}{d^{C*}}=\frac{\beta }{2\rho },$$

(A14) $$\frac{\partial (d^{C*})}{\partial \delta }=\frac{-2\beta [2\tau \rho (1-\delta )-{\left(\gamma -1\right)}^{2}\varepsilon^{2}-(\gamma -1)\alpha \varepsilon ][2\tau \rho (1-\delta )-{\left(\gamma -1\right)}^{2}\varepsilon^{2}+(\gamma -1)\alpha \varepsilon ]}{{\left\{(\delta +3)[2\tau \rho (1-\delta )-{\left(\gamma -1\right)}^{2}\varepsilon^{2}]-\alpha^{2}(1-\delta )\right\}}^{2}},$$

(A15) $$\frac{\partial (\pi_{sc}^{C*})}{\partial \delta }=\frac{\beta }{2\rho }\frac{\partial (d^{C*})}{\partial \delta },$$

(A16) $$\frac{\partial (\varsigma^{C*})}{\partial \delta }=\frac{-4\beta \alpha [-2{\left(\gamma -1\right)}^{2}\varepsilon^{2}+\tau \rho {\left(1-\delta \right)}^{2}]}{{\left\{(\delta +3)[2\rho \tau (1-\delta )-{\left(\gamma -1\right)}^{2}\varepsilon^{2}]-\alpha^{2}(1-\delta )\right\}}^{2}},$$

(A17) $$\frac{\partial (pdl^{C*})}{\partial \delta }=-m\frac{\partial (\varsigma^{C*})}{\partial \delta },$$

(A18) $$\frac{\partial (ppl^{C*})}{\partial \delta }=-\frac{2\rho m}{\beta }\frac{\partial (\varsigma^{C*})}{\partial \delta },$$

(A19) $$\frac{\partial (d^{C*})}{\partial \varepsilon }=\frac{4\beta (1-\delta )\{\alpha {\left(\gamma -1\right)}^{2}\varepsilon -(\gamma +1)[2\tau \rho (\delta -1)+{\left(\gamma -1\right)}^{2}\varepsilon^{2}]\}\alpha }{{\left\{(\delta +3)[2\rho \tau (1-\delta )-{\left(\gamma -1\right)}^{2}\varepsilon^{2}]-\alpha^{2}(1-\delta )\right\}}^{2}},$$

(A20) $$\frac{\partial (\pi_{sc}^{C*})}{\partial \varepsilon }=\frac{\beta }{2\rho }\frac{\partial (d^{C*})}{\partial \varepsilon },$$

(A21) $$\frac{\partial (\varsigma^{C*})}{\partial \varepsilon }=2(\delta -1)\beta \frac{\left(\gamma +1)[(\delta -1)(2\delta \rho \tau +6\tau +\alpha^{2})+{\left(\gamma -1\right)}^{2}\varepsilon^{2}(\delta +3)]-2\alpha {\left(\gamma -1\right)}^{2}\varepsilon (\delta +3\right)}{{\left\{(\delta +3)[2\rho \tau (1-\delta )-{\left(\gamma -1\right)}^{2}\varepsilon^{2}]-\alpha^{2}(1-\delta )\right\}}^{2}},$$

(A22) $$\frac{\partial (pdl^{C*})}{\partial \varepsilon }=-m\frac{\partial (\varsigma^{C*})}{\partial \varepsilon },$$

(A23) $$\frac{\partial (ppl^{C*})}{\partial \varepsilon }=-\frac{2\rho m}{\beta }\frac{\partial (\varsigma^{C*})}{\partial \varepsilon },$$

(A24) $$\frac{\partial (d^{C*})}{\partial \gamma }=\frac{4\beta (1-\delta )\varepsilon \alpha [2\tau \rho (1-\delta )-{\left(\gamma -1\right)}^{2}\varepsilon^{2}-(1-\gamma )\alpha \varepsilon ]}{{\left\{(\delta +3)[2\tau \rho (1-\delta )-{\left(\gamma -1\right)}^{2}\varepsilon^{2}]-\alpha^{2}(1-\delta )\right\}}^{2}},$$

(A25) $$\frac{\partial (\pi_{sc}^{C*})}{\partial \gamma }=\frac{\beta }{2\rho }\frac{\partial d^{C*}}{\partial \gamma },$$

(A26) $$\frac{\partial (\varsigma^{C*})}{\partial \gamma }=2(1-\delta )\beta \varepsilon \frac{\left(\delta +3)[2\tau \rho (1-\delta )-{\left(\gamma -1\right)}^{2}\varepsilon^{2}-2\alpha (1-\gamma )\varepsilon ]+\alpha^{2}(1-\delta \right)}{{\left\{(\delta +3)[2\tau \rho (1-\delta )-{\left(\gamma -1\right)}^{2}\varepsilon^{2}]-\alpha^{2}(1-\delta )\right\}}^{2}},$$

(A27) $$\frac{\partial (pdl^{C*})}{\partial \gamma }=-m\frac{\partial (\varsigma^{C*})}{\partial \gamma },$$

(A28) $$\frac{\partial (ppl^{C*})}{\partial \gamma }=-\frac{2\rho m}{\beta }\frac{\partial (\varsigma^{C*})}{\partial \gamma }\;\;,$$

(A29) $$\frac{\partial (d^{C*})}{\partial m}=\frac{2\rho c_{sm}[2\rho (1-\delta )\tau -{\left(\gamma -1\right)}^{2}\varepsilon^{2}]\{(\delta +3)[{\left(\gamma -1\right)}^{2}\varepsilon^{2}-2\rho \tau (1-\delta )]+\alpha^{2}(1-\delta )+4\alpha \beta (1-\delta )\}}{{\left\{(\delta +3)[2\tau \rho (1-\delta )-{\left(\gamma -1\right)}^{2}\varepsilon^{2}]-\alpha^{2}(1-\delta )\right\}}^{2}},$$

(A30) $$\frac{\partial (\pi_{sc}^{C*})}{\partial m}=\frac{2\beta c_{sm}[2(1-\delta )\rho \tau -{\left(\gamma -1\right)}^{2}\varepsilon^{2}]\{(\delta +3)[{\left(\gamma -1\right)}^{2}\varepsilon^{2}-2\tau \rho (1-\delta )]+\alpha^{2}(1-\delta )+2\alpha \beta (1-\delta )\}}{{\left\{(\delta +3)[2\tau \rho (1-\delta )-{\left(\gamma -1\right)}^{2}\varepsilon^{2}]-\alpha^{2}(1-\delta )\right\}}^{2}},$$

(A31) $$\frac{\partial (\varsigma^{C*})}{\partial m}=2(\delta -1)\rho c_{sm}\frac{\{(\delta +3)[{\left(\gamma -1\right)}^{2}\varepsilon^{2}-2\tau \rho (1-\delta )]+\alpha^{2}(1-\delta )\}(2\beta -\alpha )-4(1-\delta )\beta \alpha^{2}}{{\left\{(\delta +3)[2\tau \rho (1-\delta )-{\left(\gamma -1\right)}^{2}\varepsilon^{2}]-\alpha^{2}(1-\delta )\right\}}^{2}},$$

(A32) $$\begin{array}{l} \frac{\partial (d^{C*})}{\partial \rho }=\frac{2(\beta -1)\{(1-\delta )[2(3+\delta )\rho \tau -\alpha^{2}]-{\left(1-\gamma \right)}^{2}\varepsilon^{2}(\delta +3)-2(1-\delta )\alpha \beta \}[2\rho \tau (1-\delta )-{\left(1-\gamma \right)}^{2}\varepsilon^{2}]}{\rho {\left\{(1-\delta )[2(3+\delta )\rho \tau -\alpha^{2}]-{\left(1-\gamma \right)}^{2}\varepsilon^{2}(\delta +3)\right\}}^{2}}\\ +\frac{-4\alpha \beta (1-\delta )\{\rho (\omega +c_{l})[2\rho \tau (1-\delta )-{\left(1-\gamma \right)}^{2}\varepsilon^{2}]+\rho \varepsilon [\varepsilon mc_{sm}{\left(1-\gamma \right)}^{2}+(1-\delta )\tau (1+\gamma )]\}}{\rho {\left\{(1-\delta )[2(3+\delta )\rho \tau -\alpha^{2}]-{\left(1-\gamma \right)}^{2}\varepsilon^{2}(\delta +3)\right\}}^{2}} \end{array}$$

(A33) $$\frac{\partial (\pi_{sc}^{C*})}{\partial \rho }=\frac{\beta }{2\rho }\frac{\partial d^{C*}}{\partial \rho }-\frac{d^{C*}}{2\rho^{2}},$$

(A34) $$\begin{array}{l} \frac{\partial (\varsigma^{C*})}{\partial \rho }=2(\delta -1)\frac{\left[(1+\gamma )\varepsilon \beta +(1-\beta )\alpha ]\{(1-\delta )[2(3+\delta )\rho \tau -\alpha^{2}]-{\left(1-\gamma \right)}^{2}\varepsilon^{2}(\delta +3)-2(1-\delta )\alpha \beta \right\}}{\rho {\left\{(1-\delta )[2(3+\delta )\rho \tau -\alpha^{2}]-{\left(1-\gamma \right)}^{2}\varepsilon^{2}(\delta +3)\right\}}^{2}}\\ +\frac{2(\delta -1)\{\alpha \beta {\left(1-\gamma \right)}^{2}\varepsilon^{2}(\delta +3)+\alpha \beta (1-\delta )[(1+\gamma )(2\beta +\alpha )\varepsilon +2\rho \alpha (\omega +c_{l})]\}}{\rho {\left\{(1-\delta )[2(3+\delta )\rho \tau -\alpha^{2}]-{\left(1-\gamma \right)}^{2}\varepsilon^{2}(\delta +3)\right\}}^{2}} \end{array}$$

(A35) $$\frac{\partial (pdl^{C*})}{\partial \rho }=-m\frac{\partial (\varsigma^{C*})}{\partial \rho },$$

(A36) $$\frac{\partial (ppl^{C*})}{\partial \rho }=-\frac{2\rho m}{\beta }\frac{\partial (\varsigma^{C*})}{\partial \rho }+(1-\varsigma^{C*})m\frac{2}{\beta }.$$

From Equations (B*_i_*) (*i* = 4, 5…, 26, 27), we can get the following results in the centralized model under the conditions of *L_i_ (i = A, B*): (1) the marginal profit of FASC is $\frac{\beta }{2\rho }$; (2) $\frac{\partial (d^{C*})}{\partial \delta }<0$ and $\frac{\partial (\pi_{sc}^{C*})}{\partial \delta }<0$; (3) if ${\left(1-\delta \right)}^{2}\tau \rho >2{\left(\gamma -1\right)}^{2}\varepsilon^{2}$, then $\frac{\partial (\varsigma^{C*})}{\partial \delta }<0$, $\frac{\partial (pdl^{C*})}{\partial \delta }>0$ and $\frac{\partial (ppl^{C*})}{\partial \delta }>0$; otherwise, $\frac{\partial (\varsigma^{C*})}{\partial \delta }>0$, $\frac{\partial (pdl^{C*})}{\partial \delta }<0$ and $\frac{\partial (ppl^{C*})}{\partial \delta }<0$; (4) $\frac{\partial (d^{C*})}{\partial \varepsilon }>0$, $\frac{\partial (\pi_{sc}^{C*})}{\partial \varepsilon }>0$, $\frac{\partial (\varsigma^{C*})}{\partial \varepsilon }>0$, $\frac{\partial (pdl^{C*})}{\partial \varepsilon }<0$ and $\frac{\partial (ppl^{C*})}{\partial \varepsilon }<0$; (5) $\frac{\partial (d^{C*})}{\partial \gamma }>0$ and $\frac{\partial (\pi_{sc}^{C*})}{\partial \gamma }>0$; (6) if $(\delta +3)[2\tau \rho (1-\delta )-2\alpha (1-\gamma )\varepsilon -{\left(\gamma -1\right)}^{2}\varepsilon^{2}]+\alpha^{2}(1-\delta )<0$, $\frac{\partial \varsigma^{C*}}{\partial \gamma }<0$, $\frac{\partial (pdl^{C*})}{\partial \gamma }>0$ and $\frac{\partial (ppl^{C*})}{\partial \gamma }>0$; otherwise, $\frac{\partial (\varsigma^{C*})}{\partial \gamma }>0$, $\frac{\partial (pdl^{C*})}{\partial \gamma }<0$ and $\frac{\partial (ppl^{C*})}{\partial \gamma }<0$; (6) if $(\delta +3)[{\left(\gamma -1\right)}^{2}\varepsilon^{2}-2\tau \rho (1-\delta )]+\alpha^{2}(1-\delta )+4\alpha \beta (1-\delta )>0$, $\frac{\partial (d^{C*})}{\partial m}>0$; otherwise, $\frac{\partial (d^{C*})}{\partial m}<0$; (8) $\frac{\partial (\pi_{sc}^{C*})}{\partial m}<0$; (9) if $\{(\delta +3)[{\left(\gamma -1\right)}^{2}\varepsilon^{2}-2\tau \rho (1-\delta )]+\alpha^{2}(1-\delta )\}(2\beta -\alpha )-4(1-\delta )\beta \alpha^{2}<0$, then $\frac{\partial (\varsigma^{C*})}{\partial m}>0$; otherwise, $\frac{\partial (\varsigma^{C*})}{\partial m}<0$; (10) $\frac{\partial (d^{C*})}{\partial \rho }<0$, $\frac{\partial (\pi_{sc}^{C*})}{\partial \rho }<0$, $\frac{\partial (\varsigma^{C*})}{\partial \rho }<0$, $\frac{\partial (pdl^{C*})}{\partial \rho }>0$, $\frac{\partial (ppl^{C*})}{\partial \rho }>0$. □

**Proof of Proposition** **A4.** The Hessian matrix of $\pi_{r}^{cc}(p_{1}^{cc},p_{2}^{cc})$ about $\left(p_{1}^{cc},p_{2}^{cc}\right)$ is $\left|\begin{array}{cc} \frac{2\rho \lambda }{\delta -1} & \frac{1+\delta }{1-\delta }\rho \lambda \\ \frac{1+\delta }{1-\delta }\rho \lambda & \frac{2\rho \lambda }{\delta -1} \end{array}\right|=\frac{3+\delta }{1-\delta }\rho^{2}\lambda^{2}>0$, we can get $\frac{\partial^{2}\pi_{r}^{tr}}{\partial {\left(p_{{}_{1}}^{tr}\right)}^{2}}=\frac{\partial^{2}\pi_{r}^{tr}}{\partial {\left(p_{{}_{2}}^{tr}\right)}^{2}}<0$. So $\pi_{r}^{cc}(p_{1}^{cc},p_{2}^{cc})$ is a strictly differentiable concave function of $\left(p_{1}^{cc},p_{2}^{cc}\right)$. The optimal price in two periods are arrived at after solving the first-order condition $\left\{ \begin{array}{l} \frac{\partial \pi_{r}^{cc}}{\partial p_{1}}=0\\ \frac{\partial \pi_{r}^{cc}}{\partial p_{2}}=0 \end{array}\right.$, then we can obtain:

(A37) $$\left\{ \begin{array}{l} p_{1}^{cc}(\varsigma^{cc})=\frac{[2+(1+\gamma )\varsigma^{cc}]\lambda +(1+\delta )[(1-\varsigma^{cc})c_{sm}m+p_{l}^{cc}+\omega ]\rho }{(\delta +3)\lambda \rho }\\ p_{2}^{cc}(\varsigma^{cc})=\frac{[\delta +\varepsilon \varsigma^{cc}(-1+2\gamma +\delta \gamma )+1]\lambda +2[(1-\varsigma^{cc})c_{sm}m+p_{l}^{cc}+\omega ]\rho }{(\delta +3)\lambda \rho } \end{array}\right..$$

Substitute Equation (A37) into the profit function of TPLSP, we can get that $\pi_{l}^{cc}(\varsigma^{cc})$ is a strictly differentiable concave function of $\varsigma^{cc}$ if $\frac{\partial^{2}\pi_{l}^{cc}}{\partial {\left(\varsigma^{cc}\right)}^{2}}=\frac{\left\{2\varepsilon^{2}[(\delta +1)\gamma -1-\gamma^{2}]\lambda^{2}+2(1-\delta )\rho^{2}c_{sm}^{2}m^{2})\}(\lambda -1)-\lambda^{2}\mu \rho \tau (1-\delta )(\delta +3\right)}{(1-\delta )(\delta +3)\rho \lambda^{2}}<0$ is satisfied. The optimal logistics service level is arrived at after solving the first-order condition $\frac{\partial \pi_{l}^{cc}}{\partial \varsigma^{cc}}=0$, then we can obtain:

(A38) $$\varsigma^{cc*}(\lambda ,\mu ,p_{l}^{cc})=\frac{\left(1-\delta )\{\varepsilon (1+\gamma )\lambda^{2}[\lambda +(c_{l}-p_{l}^{cc})\rho -1]+2\rho^{2}mc_{sm}[(mc_{sm}+\omega )(\lambda -1)+c_{l}\lambda -p_{l}^{cc}]\right\}}{\left\{2\varepsilon^{2}[(\delta +1)\gamma -1-\gamma^{2}]\lambda^{2}+2\rho^{2}m^{2}c_{sm}^{2}(1-\delta )\}(\lambda -1)-\lambda^{2}\mu \rho \tau (1-\delta )(\delta +3\right)}.$$

Substitute Equation (A38) into the Equation (A37), we can get $p_{1}^{cc*}(\lambda ,\mu ,p_{l}^{cc})$ and $p_{2}^{cc*}(\lambda ,\mu ,p_{l}^{cc})$. In order to coordinate the supply chain, the optimal decisions in contractual cooperation model are equal to those in centralized model. Let $\left\{ \begin{array}{l} p_{1}^{cc*}(\lambda ,\mu ,p_{l}^{cc})=p_{1}^{C*}\\ p_{2}^{cc*}(\lambda ,\mu ,p_{l}^{cc})=p_{2}^{C*}\\ \varsigma^{cc*}=\varsigma^{C*} \end{array}\right.$, then we can get:

(A39) $$\left\{ \begin{array}{l} 0<\lambda^{*}<1\\ \mu^{*}=\frac{\varepsilon [mc_{sm}{\left(\gamma -1\right)}^{2}\varepsilon +\tau (\gamma +1)(1-\delta )](1-\lambda^{*})}{(1-\delta )\alpha \tau }\\ p_{l}^{cc*}=c_{l}-(1-\lambda^{*})\frac{\left\{(\delta +3)[2(1-\delta )\tau \rho -\varepsilon^{2}{\left(\gamma -1\right)}^{2}]-(1-\delta )\alpha^{2}-2(1-\delta )\alpha \beta \}(1-\beta )+2(1-\delta )\alpha \beta (\omega +c_{l}\right)}{\{(\delta +3)[2(1-\delta )\tau \rho -\varepsilon^{2}{\left(\gamma -1\right)}^{2}]-(1-\delta )\alpha^{2}\}\rho } \end{array}\right.$$

Under the conditions of *L_i_ (i = A,B)*, $0<\mu^{*}<1$ and $\frac{\partial^{2}\pi_{l}^{cc}}{\partial {\left(\varsigma^{cc}\right)}^{2}}<0$ are satisfied. Substitute Equation (A39) into the profit functions of the TPLSP and the retailer, we can get $\left\{ \begin{array}{l} \pi_{r}^{cc*}=\frac{\beta^{2}[{\left(\gamma -1\right)}^{2}\varepsilon^{2}+2(\delta -1)\rho \tau ]\{(\delta +3){\left(\gamma -1\right)}^{2}\varepsilon^{2}\lambda^{*}-\alpha (\delta -1)(\lambda^{*}-1)(\gamma +1)\varepsilon +[2\rho \tau \lambda^{*}(\delta +3)-\alpha^{2}](\delta -1)\}}{\rho {\left[(\delta +3){\left(\gamma -1\right)}^{2}\varepsilon^{2}+(2\rho \tau \delta -\alpha^{2}+6\rho \tau )(\delta -1)\right]}^{2}}\\ \pi_{l}^{cc*}=\frac{\beta^{2}[{\left(\gamma -1\right)}^{2}\varepsilon^{2}+2(\delta -1)\rho \tau ][(\delta +3){\left(\gamma -1\right)}^{2}\varepsilon^{2}-\alpha (\delta -1)(\gamma +1)\varepsilon +2\rho \tau (\delta +3)(\delta -1)](1-\lambda^{*})}{\rho {\left[(\delta +3){\left(\gamma -1\right)}^{2}\varepsilon^{2}+(2\rho \tau \delta -\alpha^{2}+6\rho \tau )(\delta -1)\right]}^{2}} \end{array}\right.$ and $\pi_{r}^{cc*}+\pi_{l}^{cc*}=\pi_{sc}^{C*}$. So the coordination of FASC can be realized with the revenue and service-cost sharing contract. If $\left\{ \begin{array}{l} 1-N\frac{\left(\delta +3)[2(1-\delta )\tau \rho -\varepsilon^{2}{\left(\gamma -1\right)}^{2}]-\alpha^{2}(1-\delta \right)}{M[4(\delta +3)\rho \tau -\alpha^{2}](1-\delta )}<\lambda^{*}\\ \lambda^{*}<1-\tau \rho \frac{2{\left\{(\delta +3)[2(1-\delta )\tau \rho -\varepsilon^{2}{\left(\gamma -1\right)}^{2}]-(1-\delta )\alpha^{2}\right\}}^{2}}{M} \end{array}\right.$, where $M=\{(\delta +3)[2(1-\delta )\tau \rho -\varepsilon^{2}{\left(\gamma -1\right)}^{2}]-(1-\delta )\alpha (1+\gamma )\varepsilon \}[2(1-\delta )\tau \rho -\varepsilon^{2}{\left(\gamma -1\right)}^{2}][4(\delta +3)\tau \rho -\alpha^{2}]$, $N=6(\delta +3)[2\tau \rho (1-\delta )-\varepsilon^{2}{\left(\gamma -1\right)}^{2}](1-\delta )[2(\delta +3)\rho \tau -\alpha^{2}]+\alpha^{2}\varepsilon^{4}{\left(\gamma -1\right)}^{4}(\delta +3)+2\tau \rho {\left(\delta -1\right)}^{2}\alpha^{4}$ and $\begin{array}{l} 1-\tau \rho \frac{2{\left\{(\delta +3)[2(1-\delta )\tau \rho -\varepsilon^{2}{\left(\gamma -1\right)}^{2}]-(1-\delta )\alpha^{2}\right\}}^{2}}{M}-\{1-N\frac{\left(\delta +3)[2(1-\delta )\tau \rho -\varepsilon^{2}{\left(\gamma -1\right)}^{2}]-\alpha^{2}(1-\delta \right)}{M[4(\delta +3)\rho \tau -\alpha^{2}](1-\delta )}\}\\ =\frac{(\delta +3)\{(\delta +3)[2\rho (1-\delta )\tau -\varepsilon^{2}{\left(\gamma -1\right)}^{2}]-(1-\delta )\alpha^{2}\}\{\alpha^{2}\varepsilon^{4}{\left(\gamma -1\right)}^{4}+\rho \tau (1-\delta )[8\rho^{2}\tau^{2}(1-\delta )(3+\delta )-2\varepsilon^{2}{\left(\gamma -1\right)}^{2}(\delta +3)\tau \rho +2\varepsilon^{2}{\left(\gamma -1\right)}^{2}\alpha^{2}]\}}{M[4\rho (\delta +3)\tau -\alpha^{2}](1-\delta )}>0 \end{array}$, we can obtain that $\lambda^{*}$ $\left(\lambda^{*}\in (0,1)\right)$ exists and $\left\{ \begin{array}{l} \pi_{r}^{cc*}-\pi_{r}^{tr*}>0\\ \pi_{l}^{cc*}-\pi_{l}^{tr*}>0 \end{array}\right.$. Then the contract $\left(\lambda^{*},\mu^{*},p_{l}^{cc*}\right)$ can reach Pareto improvement. □

**Proof of Corollary** **A1.** $\varsigma^{C*}-\varsigma^{tr*}=\frac{2(\delta +3)\alpha \beta [2(1-\delta )\rho \tau +{\left(\gamma -1\right)}^{2}\varepsilon^{2}]}{\left[4(\delta +3)\rho \tau -\alpha^{2}]\{(1-\delta )[2(\delta +3)\rho \tau -\alpha^{2}]-(\delta +3){\left(\gamma -1\right)}^{2}\varepsilon^{2}\right\}}>0$, so $\varsigma^{cc*}=\varsigma^{C*}>\varsigma^{tr*}$. Because *L_A_* and $\begin{array}{l} \left(\delta +3){\left(\gamma -1\right)}^{2}\varepsilon^{2}(\beta -1)-\beta \alpha (\delta -1)(\gamma +1)\varepsilon +(1-\delta )[2(\delta +3)(1-\beta )\rho \tau -\alpha^{2}\right]\\ =\{(1-\delta )[2(\delta +3)\rho \tau -\alpha^{2}]-(\delta +3){\left(\gamma -1\right)}^{2}\varepsilon^{2}-2(1-\delta )\alpha \beta \}(1-\beta )+2(1-\delta )\alpha \beta (c_{l}+\omega )>0 \end{array}$, we can get $p_{l}^{cc*}-c_{l}=(\lambda^{*}-1)\frac{\left\{(\delta +3){\left(\gamma -1\right)}^{2}\varepsilon^{2}(\beta -1)-\beta \alpha (\delta -1)(\gamma +1)\varepsilon +(1-\delta )[2(\delta +3)(1-\beta )\rho \tau -\alpha^{2}]\right\}}{(1-\delta )[2(\delta +3)\rho \tau -\alpha^{2}]-(\delta +3){\left(\gamma -1\right)}^{2}\varepsilon^{2}}<0$. While $p_{l}^{tr*}=c_{l}+\frac{2\tau \beta (\delta +3)}{4\rho \tau (3+\delta )-\alpha^{2}}>c_{l}$, we can obtain $p_{l}^{cc*}<c_{l}<p_{l}^{tr*}$. Under the conditions of *L_i_ (i = A,B,C,D)*, we can obtain $d^{C*}-d^{tr*}=\frac{2\beta \{4\rho^{2}\tau^{2}(1-\delta )(\delta +3)-[2(\delta +3)\rho \tau -\alpha^{2}]{\left(\gamma -1\right)}^{2}\varepsilon^{2}\}}{\left[4(\delta +3)\rho \tau -\alpha^{2}]\{2(1-\delta )[(\delta +3)\rho \tau -\alpha^{2}]-(\delta +3){\left(\gamma -1\right)}^{2}\varepsilon^{2}\right\}}>0$, so $d^{C*}-d^{tr*}>0$. We can obtain $\pi_{l}^{tr*}/d^{tr*}=\beta /\left(2\rho \right)$ from the demand and profit functions in the decentralized model and $\pi^{cc*}/d^{cc*}=\pi^{C*}/d^{C*}=\beta /\left(2\rho \right)$ from Proposition 2, so $\left(\pi_{r}^{tr*}+\pi_{l}^{tr*}\right)/d^{tr*}>\pi^{cc*}/d^{cc*}$. □

**Proof of Corollary** **A2.** From the corollary 1 we can get $ppl^{cc*}-pdl^{tr*}=m(1-\varsigma^{cc*})-m(1-\varsigma^{tr*})<0$, so $pdl^{cc*}=pdl^{C*}<pdl^{tr*}$. Since $\begin{array}{l} ppl^{tr*}-ppl^{cc*}=\\ -2\rho m\frac{{\left(\gamma -1\right)}^{4}\varepsilon^{4}\alpha^{2}(\delta +3)+[\alpha^{3}(\alpha +2\beta )+4\rho^{2}\tau^{2}{\left(\delta +3\right)}^{2}+2\alpha (2\beta -\alpha )\rho \tau (\delta +3)](1-\delta ){\left(\gamma -1\right)}^{2}\varepsilon^{2}-4\rho^{2}\tau^{2}(\delta +3){\left(1-\delta \right)}^{2}[-\alpha (\alpha +4\beta )+2\rho \tau (\delta +3)]}{\{(\delta +3)[2\tau \rho (1-\delta )-{\left(\gamma -1\right)}^{2}\varepsilon^{2}]-\alpha^{2}(1-\delta )\}\{[{\left(\gamma -1\right)}^{2}\varepsilon^{2}\alpha^{2}+2\rho \tau (1-\delta )[6(\delta +3)\rho \tau -\alpha^{2}]\}\beta } \end{array}$ and $tdl^{tr*}-tdl^{cc*}=-m\rho \frac{4\beta \tau [4(\delta +3)\tau -\alpha^{2}-2\alpha \beta ]}{{\left[\alpha^{2}-4(\delta +3)\rho \tau \right]}^{2}}+2\beta m\frac{\left[2\rho \tau (1-\delta )-{\left(\gamma -1\right)}^{2}\varepsilon^{2}]\{(1-\delta )[2(\delta +3)\rho \tau -\alpha^{2}-2\beta \alpha ]-{\left(\gamma -1\right)}^{2}\varepsilon^{2}(\delta +3)\right\}}{{\left\{(\delta +3)[2\rho \tau (1-\delta )-{\left(\gamma -1\right)}^{2}\varepsilon^{2}]-\alpha^{2}(1-\delta )\right\}}^{2}}$, if $mc>mc_{1}$, we can obtain $ppl^{tr*}-ppl^{cc*}<0$ and $tdl^{tr*}-tdl^{cc*}<0$. If $mc_{2}<mc<mc_{1}$, we can obtain $ppl^{tr*}-ppl^{cc*}>0$ and $tdl^{tr*}-tdl^{cc*}<0$. If

$$mc_{3}<mc<mc_{2},$$

we obtain $ppl^{tr*}-ppl^{cc*}>0$ and $tdl^{tr*}-tdl^{cc*}>0$. □

**Proof of Corollary** **A3.** From the function of $mc_{3}$, we can obtain:

(A40) $$\frac{\partial (mc_{3})}{\partial \delta }=\frac{2{\left(1-\gamma \right)}^{2}\varepsilon^{2}-{\left(1-\delta \right)}^{2}\rho \tau }{{\left(1-\delta \right)}^{2}\alpha \rho },$$

(A41) $$\frac{\partial (mc_{3})}{\partial \varepsilon }=\frac{-(\delta +3)\varepsilon [4\rho mc_{sm}{\left(1-\gamma \right)}^{2}+\varepsilon (1-\gamma +2\gamma^{3})]-(1+\gamma )[(1-\delta )(6\rho \tau +2\delta \rho \tau +\alpha^{2})-{\left(1-\gamma \right)}^{2}(\delta +3)\varepsilon^{2}]}{2\rho (\delta -1)\alpha^{2}}>0,$$

(A42) $$\frac{\partial (mc_{3})}{\partial \gamma }=\frac{\varepsilon \{(\delta +3)[2(1-\delta )\rho \tau -2\alpha (1-\gamma )\varepsilon -{\left(1-\gamma \right)}^{2}\varepsilon^{2}]+(1-\delta )\alpha^{2}\}}{2\rho (1-\delta )\alpha^{2}},$$

(A43) $$\frac{\partial (mc_{3})}{\partial m}=\frac{c_{sm}\{(\delta +3)[2(1-\delta )\rho \tau -{\left(1-\gamma \right)}^{2}\varepsilon^{2}]+2(1-\delta )\alpha^{2}\}}{\rho (1-\delta )\alpha^{2}},$$

(A44) $$\frac{\partial (mc_{3})}{\partial \rho }=\frac{2mc_{sm}\rho \{(\delta +3)[2(1-\delta )\rho \tau -{\left(1-\gamma \right)}^{2}\varepsilon^{2}]-(1-\delta )\alpha^{2}\}-(1-\delta )\alpha^{2}[(1+\gamma )\varepsilon +2(1-mc_{sm}\rho )]-\alpha {\left(1-\gamma \right)}^{2}\varepsilon^{2}(\delta +3)}{2\rho^{2}(1-\delta )\alpha^{2}}.$$

So $\frac{\partial (mc_{3})}{\partial \varepsilon }>0$ and $\frac{\partial (mc_{3})}{\partial m}>0$. If ${\left(1-\delta \right)}^{2}\rho \tau >2{\left(1-\gamma \right)}^{2}\varepsilon^{2}$, we can obtain $\frac{\partial (mc_{3})}{\partial \delta }<0$; otherwise, we obtain $\frac{\partial (mc_{3})}{\partial \delta }>0$. If $(\delta +3)[2\rho \tau (1-\delta )-2\alpha (1-\gamma )\varepsilon -{\left(\gamma -1\right)}^{2}\varepsilon^{2}]+\alpha^{2}(1-\delta )<0$, we can obtain $\frac{\partial (mc_{3})}{\partial \gamma }<0$, otherwise, we obtain $\frac{\partial (mc_{3})}{\partial \gamma }>0$. If $0<2mc_{sm}\rho \{(\delta +3)[2(1-\delta )\rho \tau -{\left(1-\gamma \right)}^{2}\varepsilon^{2}]-(1-\delta )\alpha^{2}\}-(1-\delta )\alpha^{2}[(1+\gamma )\varepsilon +2(1-mc_{sm}\rho )]-\alpha {\left(1-\gamma \right)}^{2}\varepsilon^{2}(\delta +3)$, we can obtain $\frac{\partial (mc_{3})}{\partial \rho }>0$; otherwise, we obtain $\frac{\partial (mc_{3})}{\partial \rho }<0$. □
